# α-Ketoheterocycles Able to Inhibit the Generation of Prostaglandin E_2_ (PGE_2_) in Rat Mesangial Cells

**DOI:** 10.3390/biom11020275

**Published:** 2021-02-13

**Authors:** Anastasia Psarra, Maria A. Theodoropoulou, Martin Erhardt, Marina Mertiri, Christiana Mantzourani, Sofia Vasilakaki, Victoria Magrioti, Andrea Huwiler, George Kokotos

**Affiliations:** 1 Department of Chemistry, National and Kapodistrian University of Athens, Panepistimiopolis, 15771 Athens, Greece; apsarra@chem.uoa.gr (A.P.); martheod@chem.uoa.gr (M.A.T.); mertmarina@gmail.com (M.M.); chrmantz@chem.uoa.gr (C.M.); svasilak@chem.uoa.gr (S.V.); vmagriot@chem.uoa.gr (V.M.); 2 Institute of Pharmacology, University of Bern, CH-3010 Bern, Switzerland; martin.erhardt@pki.unibe.ch (M.E.); huwiler@pki.unibe.ch (A.H.)

**Keywords:** anti-inflammatory, inhibition, α-ketobenzothiazoles, mesangial cells, prostaglandin E_2_

## Abstract

Prostaglandin E_2_ (PGE_2_) is a key mediator of inflammation, and consequently huge efforts have been devoted to the development of novel agents able to regulate its formation. In this work, we present the synthesis of various α-ketoheterocycles and a study of their ability to inhibit the formation of PGE_2_ at a cellular level. A series of α-ketobenzothiazoles, α-ketobenzoxazoles, α-ketobenzimidazoles, and α-keto-1,2,4-oxadiazoles were synthesized and chemically characterized. Evaluation of their ability to suppress the generation of PGE_2_ in interleukin-1β plus forskolin-stimulated mesangial cells led to the identification of one α-ketobenzothiazole (GK181) and one α-ketobenzoxazole (GK491), which are able to suppress the PGE_2_ generation at a nanomolar level.

## 1. Introduction

**Figure 1:**
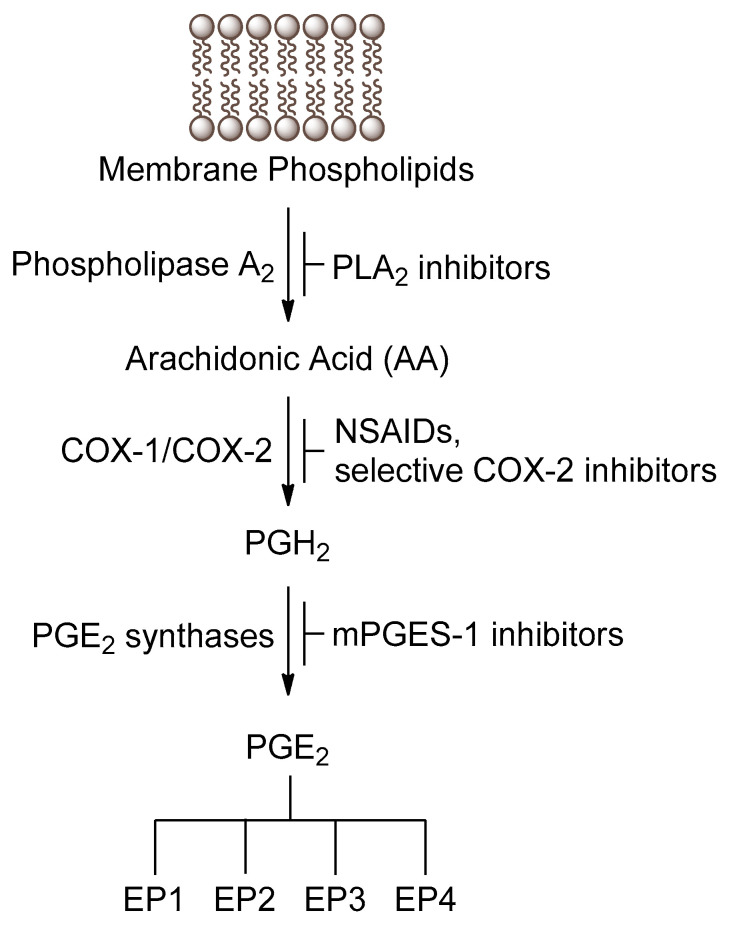
Generation of PGE_2_ through metabolism of arachidonic acid.

Prostaglandins are a class of highly bioactive eicosanoids, which are generated from arachidonic acid by the subsequent action of various enzymes [1,2]. Among them, prostaglandin E_2_ (PGE_2_) is the most abundant in humans, playing physiological and pathological roles [3]. The biosynthesis of PGE_2_ begins when phospholipase A_2_ (PLA_2_) hydrolyzes membrane glycerophospholipids to release free fatty acids, including arachidonic acid (Figure 1) [4]. Then, arachidonic acid is converted to prostaglandin H_2_ (PGH_2_) by the enzymes cyclooxygenase-1 (COX-1) and cyclooxygenase-2 (COX-2) [1]. Finally, prostaglandin synthases, such as microsomal prostaglandin E synthase-1 (mPGES-1), catalyze the generation of PGE_2_ [5,6], which exerts its actions interacting with PGE_2_ receptors (Figure 1).

PGE_2_ is a key mediator of inflammation [7,8], and consequently huge efforts have been devoted to the discovery of agents able to inhibit its production [9]. The involvement of PGE_2_ in tumorigenesis and cancer is well described in recent review articles [10,11,12]. A wide variety of inhibitors targeting the various enzymes involved in the PGE_2_ biosynthesis have been developed both in pharmaceutical industry and in academia. Inhibitors of PLA_2_ targeting the release of arachidonic acid have been described, but none of them reached the market [13]. Numerous clinically validated COX-1 and COX-2 inhibitors are known. Non-steroidal anti-inflammatory drugs (NSAIDs) are non-selective COX inhibitors, while selective COX-2 inhibitors, such as celecoxib, overcome the gastrointestinal side effects of COX-1 inhibitors, however exhibiting potential cardiovascular toxicity [14]. Although mPGES-1 inhibitors have been proposed as safer alternatives to COX-2 inhibitors, lackingcardiovascular toxicity, further research is needed so that such inhibitors enter clinical practice [5,6].

**Figure 2:**
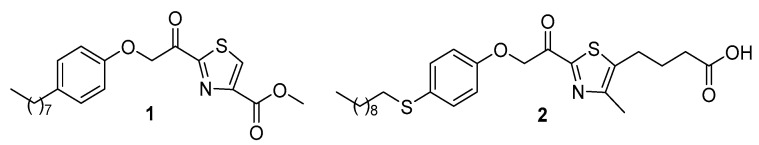
Examples of α-ketothiazoles exhibiting anti-inflammatory properties.

Sometimes, although an inhibitor for one particular enzyme involved in PGE_2_ generation presents high potency in vitro, tremendous discrepancies can be observed when it is studied in cells. Thus, we have focused our attention on evaluating potential anti-inflammatory compounds in a cellular system consisting of renal mesangial cells. We have previously shown that inhibitors of secreted PLA_2_ exhibit interesting suppression of the production of PGE_2_ in mesangial cells [15], while small peptides were also found to inhibit the generation of PGE_2_ [16]. Inspired by the anti-inflammatory properties which α-keto-thiazoles **1** and **2** (Figure 2) and related compounds exhibit [17,18,19,20], we synthesized various α-ketoheterocycles and studied their ability to inhibit the generation of PGE_2_ at a cellular level. We present herein the synthesis of a number of α-ketobenzothiazoles and related heterocycles, and we demonstrate that two of them exhibit potent suppression of the generation of PGE_2_ in rat mesangial cells.

## 2. Materials and Methods

### 2.1. General Chemistry Methods

Chromatographic purification of products was accomplished using forced-flow chromatography on Merck^®^ (Merck, Darmstadt, Germany) Kieselgel 60 F_254_ 230–400 mesh. Thin-layer chromatography (TLC) was performed on aluminum-backed silica plates (0.2 mm, 60 F_254_). Visualization of the developed chromatogram was performed by fluorescence quenching using phosphomolybdic acid, ninhydrin, or potassium permagnate stains. Melting points were determined on a Buchi^®^ 530 (Buchi, Flawil, Switzerland) spectrometer and were uncorrected. ^1^H and ^13^C NMR spectra were recorded on a Varian^®^ Mercury (Varian, Palo Alto, CA, USA) (200 MHz and 50 MHz, respectively), a Bruker Avance Neo (400 MHz and 100 MHz, respectively) (Bruker, Faellanden, Switzerland), or a Bruker Avance (500 MHz and 125 MHz, respectively) (Bruker, Santa Barbara, CA, USA), and are internally referenced to residual solvent signals. Data for ^1^H NMR are reported as follows: chemical shift (δ ppm), multiplicity (s = singlet, d = doublet, t = triplet, m = multiplet, br s = broad signal), coupling constant, integration, and peak assignment. Data for ^13^C NMR are reported in terms of chemical shift (δ ppm). IR spectra were recorded with an OSTEC, IROS-05, FTIR spectrophotometer equipped with ATR diamond crystal (Simex Co., Ltd., Nizhny Novgorod, Russia). Mass spectra (ESI) were recorded on a Finnigan^®^ Surveyor MSQ LC-MS spectrometer (Thermo, Darmstadt, Germany). High-resolution mass spectrometry (HRMS) spectra were recorded on a Bruker^®^ Maxis Impact QTOF (Bruker Daltonics, Bremen, Germany) spectrometer. A microwave synthesizer, Discover (CEM, Charlotte, NC, USA), was used for the microwave synthesis. ^1^H NMR and ^13^C NMR spectra of the final products are shown in the Supplementary Materials.

Compounds **18**, **19**, **20a**, **21a**, and **22a** were synthesized as previously described [21], and their analytical data were in accordance with literature.

General procedure for the synthesis of Weinreb amides **4a**–**f** from carboxylic acids.

To a stirred solution of the carboxylic acid **3a**–**f** (1 mmol) in dry CH_2_Cl_2_ (7 mL), 4-dimethylaminopyridine (DMAP) (1 mmol), *N*,*O*-dimethyl hydroxylamine hydrochloride (1 mmol), *N*-methylmorpholine (1 mmol), and *N*-(3-dimethylaminopropyl)-*N’*-ethyl carbodiimide hydrochloride (WSCI·HCl) (1 mmol) were added consecutively at room temperature. The reaction mixture was left stirring for 18 h. It was then washed with an aqueous solution of 10% citric acid (3 × 10 mL), brine (10 mL), an aqueous solution of 5% NaHCO_3_ (3 × 10 mL), and brine (10 mL). The organic layer was dried over Na_2_SO_4_ and concentrated under reduced pressure. The amide was purified by flash chromatography eluting with the appropriate mixture of EtOAc:petroleum ether (40–60 °C) to afford the desired product.

*N*-Methoxy-*N*-methyl-5-(naphthalen-2-yl) pentanamide (**4a**) [19]. Yield 75%; Colorless oil; ^1^H NMR (200 MHz, CDCl_3_): *δ* = 7.85–7.70 (m, 3H, 3 × ArH), 7.61 (s, 1H, ArH), 7.50–7.27 (m, 3H, 3 × ArH), 3.64 (s, 3H, OCH_3_), 3.16 (s, 3H, NCH_3_), 2.80 (t, *J* = 7.0 Hz, 2H, CH_2_), 2.45 (t, *J* = 7.0 Hz, 2H, CH_2_), 1.87–1.60 (m, 4H, 2 × CH_2_); ^13^C NMR (50 MHz, CDCl_3_): *δ* = 175.8, 139.9, 133.6, 131.9, 127.8, 127.6, 127.4, 127.3, 126.3, 125.8, 125.0, 61.2, 35.9, 31.7, 31.1, 24.4; MS (ESI) *m/z* (%): 272 [(M+H)^+^, 100].

*N*-Methoxy-5-(4-methoxyphenyl)-*N*-methylpentanamide (**4b**). Yield 64%; Colorless oil; ^1^H NMR (200 MHz, CDCl_3_): *δ* = 7.10 (d, *J* = 8.0 Hz, 2H, 2 × ArH), 6.82 (d, *J* = 8.0 Hz, 2H, 2×ArH), 3.78 (s, 3H, OCH_3_), 3.66 (s, 3H, OCH_3_), 3.17 (s, 3H, NCH_3_), 2.59 (t, *J* = 6.0 Hz, 2H, CH_2_), 2.44 (t, *J* = 6.0 Hz, 2H, CH_2_), 1.73–1.61 (m, 4H, 2 × CH_2_); ^13^C NMR (50 MHz, CDCl_3_): *δ* = 176.4, 157.5, 134.3, 129.2, 113.6, 61.1, 55.1, 34.7, 31.6, 31.4, 24.2; MS (ESI) *m/z* (%): 252.2 [(M+H)^+^, 100].

*N*-Methoxy-*N*-methyl-2-(phenethylthio)acetamide (**4c**). Yield 83%; Colorless oil; ^1^H NMR (200 MHz, CDCl_3_): *δ* = 7.31−7.10 (m, 5H, 5 × ArH), 3.63 (s, 3H, OCH_3_), 3.31 (s, 2H, SCH_2_), 3.16−3.04 (m, 4H, SCH_2_,CH_2_), 2.87 (s, 3H, NCH_3_); ^13^C NMR (50 MHz, CDCl_3_): *δ* = 159.2, 156.7, 128.1, 128.0, 125.9, 61.1, 35.3, 33.3, 31.2; HRMS (ESI) [M+Na]^+^ *m/z*: 262.0872; (calculated for [C_12_H_17_NNaO_2_S]^+^ 262.0872).

*N*-Methoxy-*N*-methyl-2-(2-(naphthalen-2-yl)ethoxy)acetamide (**4d**). Yield 72%; Colorless oil; ^1^H NMR (200 MHz, CDCl_3_): *δ* = 7.83−7.35 (m, 7H, 7 × ArH), 4.24 (s, 2H, OCH_2_), 3.85 (t, *J* = 7.1 Hz, 2H, OCH_2_), 3.52 (s, 3H, OCH_3_), 3.17−3.03 (m, 5H, NCH_3_, CH_2_); ^13^C NMR (50 MHz, CDCl_3_): *δ* = 170.7, 135.9, 133.2, 131.8, 127.6, 127.3, 127.22, 127.17, 126.9, 125.6, 125.0, 72.2, 68.1, 61.0, 36.0, 31.9; HRMS (ESI) [M+Na]^+^ *m/z*: 296.1254; (calculated for [C_16_H_19_NNaO_3_]^+^ 296.1257).

3-([1,1′-Biphenyl]-4-yl)-*N*-methoxy-*N*-methylpropanamide (**4e**). Yield 88%; Colorless oil; ^1^H NMR (200 MHz, CDCl_3_): *δ* = 7.60−7.23 (m, 9H, 9 × ArH), 3.63 (s, 3H, OCH_3_), 3.19 (s, 3H, NCH_3_), 3.00 (t, *J* = 8.1 Hz, 2H, CH_2_), 2.81 (t, *J* = 8.2 Hz, 2H, CH_2_); ^13^C NMR (50 MHz, CDCl_3_): *δ* = 140.9, 140.4, 139.0, 128.8, 128.7, 127.1, 127.0, 126.9, 61.2, 33.7, 32.2, 30.2; HRMS (ESI) [M+Na]^+^ *m/z*: 292.1308; (calculated for [C_17_H_19_NNaO_2_]^+^ 292.1308).

*N*-Methoxy-*N*-methyl-3-(naphthalen-2-yl)propanamide (**4f**). Yield 93%; Colorless oil; ^1^H NMR (200 MHz, CDCl_3_): *δ* = 7.82−7.32 (m, 7H, 7 × ArH), 3.59 (s, 3H, OCH_3_), 3.23−3.07 (m, 5H, NCH_3_, CH_2_), 2.91−2.76 (m, 2H, CH_2_); ^13^C NMR (50 MHz, CDCl_3_): *δ* = 173.4, 138.7, 133.4, 131.9, 127.9, 127.5, 127.3, 127.1, 126.4, 125.8, 125.1, 61.1, 33.5, 32.0, 30.7; HRMS (ESI) [M+Na]^+^ *m/z*: 266.1151; (calculated for [C_15_H_17_NNaO_2_]^+^ 266.1151).

General procedure for the synthesis of Weinreb amides **4g,h** from esters.

To a stirred solution of ester **5** or **6** (1 mmol) in dry tetrahydrofuran (2 mL) at −20 °C, *N*,*O*-dimethyl hydroxylamine hydrochloride (1.5 mmol) was added. Isopropyl magnesium chloride was then added dropwise over 15 min and the reaction mixture was left stirring for 35 min at −20 °C. The reaction mixture was quenched with a saturated solution of NH_4_Cl (5 mL) and the reaction mixture was extracted with diethyl ether (2 × 10 mL). The combined extracts were dried over Na_2_SO_4_ and concentrated under reduced pressure. Purification by flash chromatography eluting with the appropriate mixture of EtOAc:petroleum ether (40–60 °C) afforded the desired product.

*N*-Methoxy-*N*-methyl-2-(naphthalen-2-yloxy)acetamide (**4g**). Yield 41%; White solid; mp: 74–75 °C; ^1^H NMR (200 MHz, CDCl_3_): *δ* = 7.88–6.99 (m, 7H, 7 × ArH), 4.91 (s, 2H, CH_2_), 3.77 (s, 3H, OCH_3_), 3.25 (s, 3H, NCH_3_); ^13^C NMR (50 MHz, CDCl_3_): *δ* = 201.7, 156.2, 144.2, 134.4, 129.7, 127.7, 126.9, 126.5, 124.0, 118.9, 107.3, 65.7, 61.8, 32.5. HRMS (ESI) [M+Na]^+^ *m/z*: 268.0940; (calculated for [C_14_H_15_NNaO_3_]^+^ 268.0944).

*N*-Methoxy-2-(4-methoxyphenoxy)-*N*-methylacetamide (**4h**). Yield 52%; Colorless oil; ^1^H NMR (200 MHz, CDCl_3_): *δ* = 6.88–6.66 (m, 4H, 4 × ArH), 4.67 (s, 2H, CH_2_), 3.66 (s, 3H, OCH_3_), 3.64 (s, 3H, OCH_3_), 3.13 (s, 3H, NCH_3_); ^13^C NMR (50 MHz, CDCl_3_): *δ* = 169.3, 154.1, 152.3, 115.8, 114.4, 66.2, 61.4, 55.4, 32.1. HRMS (ESI) [M+Na]^+^ *m/z*: 248.0891; (calculated for [C_11_H_15_NNaO_4_]^+^ 248.0893.

General procedure for the synthesis of α-ketobenzothiazoles **8a**–**h**.

To a stirred solution of benzothiazole (3 mmol) in dry Et_2_O (20 mL) at −78 °C, under a dry argon atmosphere, a solution of n-BuLi (1.6 M in hexane, 3 mmol) was added dropwise over a period of 10 min. The resulting orange solution was stirred for 45 min. Then, a solution of the Weinreb amide (1 mmol) in dry Et_2_O (2 mL) was slowly added giving the mixture a dark brown color. After stirring for 30 min at −78 °C, the mixture was allowed to warm up to room temperature over a period of 2 h. Then, saturated aqueous ammonium chloride solution was added, and the reaction mixture was extracted with diethyl ether (2 × 10 mL). The combined extracts were washed with brine (10 mL) and then dried over Na_2_SO_4_ and concentrated under reduced pressure. Purification by flash chromatography eluting with the appropriate mixture of EtOAc:petroleum ether (40–60 °C) afforded the desired product.

1-(Benzo[*d*]thiazol-2-yl)-5-(naphthalen-2-yl)pentan-1-one (**8a**) [19]. Yield 72%; Yellow solid; ^1^H NMR (400 MHz, CDCl_3_): *δ* = 8.18 (d, *J* = 8.0 Hz, 1H, ArH), 7.97 (d, *J* = 8.0 Hz, 1H, ArH), 7.83−7.72 (m, 3H, 3 × ArH), 7.63 (s, 1H, ArH), 7.61−7.49 (m, 2H, 2 × ArH), 7.49−7.38 (m, 2H, 2 × ArH), 7.35 (dd, *J_1_* = 8.4, *J_2_* = 1.7 Hz, 1H, ArH), 3.34 (t, *J* = 6.9 Hz, 2H, CH_2_CO), 2.86 (t, *J* = 7.1 Hz, 2H, CH_2_Ar), 1.98–1.79 (m, 4H, 2 × CH_2_); ^13^C NMR (100 MHz, CDCl_3_): *δ* = 195.5, 166.7, 153.7, 139.8, 137.4, 133.8, 132.1, 128.0, 127.8, 127.7, 127.6, 127.5, 127.1, 126.6, 126.0, 125.5, 125.2, 122.6, 38.5, 36.0, 30.9, 23.8; IR: $\tilde{v}$ = 3052, 1687, 1601, 1551, 1490 cm^−1^; HRMS (ESI) [M+Na]^+^ *m/z*: 368.1084; (calculated for [C_22_H_19_NNaOS]^+^ 368.1080).

1-(Benzo[*d*]thiazol-2-yl)-5-(4-methoxyphenyl)pentan-1-one (**8b**). Yield 52%; Orange solid; mp: 65–66 °C; ^1^H NMR (200 MHz, CDCl_3_): *δ* = 8.18 (d, *J* = 7.6 Hz, 1H, ArH), 7.96 (d, *J* = 7.2 Hz, 1H, ArH), 7.62−7.45 (m, 2H, 2 × ArH), 7.12 (d, *J* = 8.6 Hz, 2H, 2 × ArH), 6.83 (d, *J* = 8.6 Hz, 2H, 2 × ArH), 3.77 (s, 3H, OCH_3_), 3.30 (t, *J* = 7.1 Hz, 2H, CH_2_), 2.64 (t, *J* = 7.3 Hz, 2H, CH_2_), 1.95−1.64 (m, 4H, 2 × CH_2_); ^13^C NMR (50 MHz, CDCl_3_): *δ* = 195.4, 166.5, 157.7, 153.6, 137.3, 134.2, 129.3, 127.6, 127.0, 125.4, 122.5, 113.7, 55.2, 38.4, 34.8, 31.2, 23.6; HRMS (ESI) [M+Na]^+^ *m/z*: 348.1032; (calculated for [C_19_H_19_NNaO_2_S]^+^ 348.1029).

1-(Benzo[*d*]thiazol-2-yl)-2-(phenethylthio)ethan-1-one (**8c**). Yield 86%; Orange solid; mp: 63–65 °C; ^1^H NMR (500 MHz, CDCl_3_): *δ* = 8.24 (d, *J* = 8.0 Hz, 1H, ArH), 8.03 (d, *J* = 7.8 Hz, 1H, ArH), 7.63 (t, *J* = 7.3 Hz, 1H, ArH), 7.58 (t, *J* = 7.0 Hz, 1H, ArH), 7.39−7.33 (m, 2H, 2 × ArH), 7.32−7.26 (m, 3H, 3 × ArH), 4.14 (s, 2H, SCH_2_), 3.03−2.96 (m, 4H, SCH_2_, CH_2_); ^13^C NMR (125 MHz, CDCl_3_): *δ* = 188.9, 165.4, 153.5, 140.1, 137.7, 128.62, 128.56, 127.9, 127.1, 126.5, 125.6, 122.5, 36.2, 35.7, 33.9; IR: $\tilde{v}$ = 3060, 1678, 1603, 1556 cm^−1^; HRMS (ESI) [M+Na]^+^ *m/z*: 336.0484; (calculated for [C_17_H_15_NNaOS_2_]^+^ 336.0487).

1-(Benzo[*d*]thiazol-2-yl)-2-(2-(naphthalen-2-yl)ethoxy)ethan-1-one (**8d**). Yield 20%; Orange solid; mp: 57–59 °C; ^1^H NMR (200 MHz, CDCl_3_): *δ* = 8.22−8.08 (m, 1H, ArH), 8.06−7.92 (m, 1H, ArH), 7.91−7.69 (m, 4H, 4 × ArH), 7.60−7.38 (m, 5H, 5 × ArH), 5.15 (s, 2H, OCH_2_), 3.99 (t, *J* = 7.2 Hz, 2H, OCH_2_), 3.21 (t, *J* = 7.2 Hz, 2H, CH_2_Ar); ^13^C NMR (50 MHz, CDCl_3_): *δ* = 191.2, 164.0, 153.5, 136.9, 136.0, 133.7, 132.3, 128.1, 128.0, 127.74, 127.66, 127.4, 127.3, 126.1, 125.5, 122.6, 73.7, 73.0, 36.5; IR: $\tilde{v}$ = 3058, 1708, 1634, 1589 cm^−1^; HRMS (ESI) [M+Na]^+^ *m/z*: 370.0883; (calculated for [C_21_H_17_NNaO_2_S]^+^ 370.0872).

3-([1,1′-Biphenyl]-4-yl)-1-(benzo[*d*]thiazol-2-yl)propan-1-one (**8e**). Yield 91%; Yellowish solid; mp: 44–46 °C; ^1^H NMR (400 MHz, CDCl_3_): *δ* = 8.20 (d, *J* = 8.1 Hz, 1H, ArH), 7.97 (d, *J* = 7.9 Hz, 1H, ArH), 7.65–7.50 (m, 6H, 6 × ArH), 7.46 (t, *J* = 7.6 Hz, 2H, 2 × ArH), 7.42-7.33 (m, 3H, 3 × ArH), 3.69 (t, *J* = 7.6 Hz, 2H, CH_2_), 3.22 (t, *J* = 7.6 Hz, 2H, CH_2_); ^13^C NMR (100 MHz, CDCl_3_): *δ* = 194.4, 166.2, 153.6, 141.0, 139.8, 139.2, 137.3, 129.0, 128.8, 127.7, 127.3, 127.2, 127.1, 127.0, 125.5, 122.5, 40.2, 29.4; HRMS (ESI) [M+Na]^+^ *m/z*: 366.0934; (calculated for [C_22_H_17_NNaOS]^+^ 366.0923).

1-(Benzo[*d*]thiazol-2-yl)-3-(naphthalen-2-yl)propan-1-one (**8f**). Yield 91%; Yellowish solid of low melting point; ^1^H NMR (400 MHz, CDCl_3_): *δ* = 8.20 (d, *J* = 8.2 Hz, 1H, ArH), 7.96 (d, *J* = 8.0 Hz, 1H, ArH), 7.87−7.78 (m, 3H, 3 × ArH), 7.75 (s, 1H, ArH), 7.60−7.41 (m, 5H, 5 × ArH), 3.73 (t, *J* = 7.6 Hz, 2H, CH_2_), 3.32 (t, *J* = 7.6 Hz, 2H, CH_2_); ^13^C NMR (100 MHz, CDCl_3_): *δ* = 194.4, 166.2, 153.6, 138.2, 137.3, 133.7, 132.2, 128.2, 127.71, 127.68, 127.6, 127.3, 127.0, 126.7, 126.1, 125.5, 125.4, 122.5, 40.2, 29.9; HRMS (ESI) [M+Na]^+^ *m/z*: 340.0779; (calculated for [C_20_H_15_NNaOS]^+^ 340.0767).

1-(Benzo[*d*]thiazol-2-yl)-2-(naphthalen-2-yloxy)ethan-1-one (**8g**). Yield 22%; Pale yellow solid; mp: 144–145 °C; ^1^H NMR (400 MHz, CDCl_3_): *δ* = 8.28 (d, *J* = 8.1 Hz, 1H, ArH), 8.07 (d, *J* = 7.9 Hz, 1H, ArH), 7.86−7.79 (m, 2H, 2 × ArH), 7.76 (d, *J* = 8.2 Hz, 1H, ArH), 7.70−7.59 (m, 2H, 2 × ArH), 7.50−7.44 (m, 1H, ArH), 7.42−7.34 (m, 2H, 2 × ArH), 7.28 (d, *J* = 8.9 Hz, 1H, ArH), 5.81 (s, 2H, CH_2_); ^13^C NMR (100 MHz, CDCl_3_): *δ* = 188.7, 163.6, 155.9, 153.5, 137.1, 134.3, 129.8, 129.5, 128.1, 127.7, 127.4, 126.9, 126.5, 125.6, 124.1, 122.6, 118.7, 107.6, 70.3; IR: $\tilde{v}$ = 3058, 1715, 1634, 1598, 1484 cm^-1^; HRMS (ESI) [M+Na]^+^ *m/z*: 342.0568; (calculated for [C_19_H_13_NNaO_2_S]^+^ 342.0559).

1-(Benzo[*d*]thiazol-2-yl)-2-(4-methoxyphenoxy)ethan-1-one (**8h**). Yield 27%; Pale yellow solid of low melting point; ^1^H NMR (400 MHz, CDCl_3_): *δ* = 8.20 (d, *J* = 8.0 Hz, 1H, ArH), 8.02 (d, *J* = 7.9 Hz, 1H, ArH), 7.67−7.53 (m, 2H, 2 × ArH), 6.99 (d, *J* = 8.4 Hz, 2H, 2 × ArH), 6.85 (d, *J* = 8.4 Hz, 2H, 2 × ArH), 5.61 (s, 2H, CH_2_), 3.77 (s, 3H, CH_3_); ^13^C NMR (100 MHz, CDCl_3_): *δ* = 189.5, 163.7, 154.8, 153.6, 152.3, 137.1, 128.2, 127.4, 125.6, 122.7, 116.4, 114.9, 71.5, 55.9; HRMS (ESI) [M+Na]^+^ *m/z*: 322.0514; (calculated for [C_16_H_13_NNaO_3_S]^+^ 322.0508).

*tert*-Butyl 2-(2-(naphthalen-2-yl)ethoxy)acetate (**10**). Alcohol **9** (1 mmol), *tert*-butyl bromoacetate (1.2 mmol), and Bu_4_NHSO_4_ (0.2 mmol) were diluted in toluene (1 mL) and in an aqueous solution of 50% NaOH (1 mL). The reaction mixture was stirred for 18 h. After completion of the reaction, the organic layer was collected and washed with brine (2 mL), and then dried over Na_2_SO_4_ and concentrated under reduced pressure. Purification by flash chromatography eluting with a mixture of EtOAc:petroleum ether (40–60 °C) 2:8 afforded the desired product. Yield 94%; Colorless oil; ^1^H NMR (200 MHz, CDCl_3_): *δ* = 7.90–7.31 (m, 7H, 7 × ArH), 4.01 (s, 2H, OCH_2_), 3.85 (t, *J* = 7.2 Hz, 2H, OCH_2_), 3.14 (t, *J* = 7.2 Hz, 2H, CH_2_Ar), 1.50 (s, 9H, 3 × CH_3_); ^13^C NMR (50 MHz, CDCl_3_): *δ* = 169.6, 136.0, 133.5, 132.1, 127.8, 127.5, 127.4, 127.1, 125.8, 125.2, 81.5, 72.3, 68.8, 36.3, 28.0. HRMS (ESI) [M+Na]^+^ *m/z*: 309.1458; (calculated for [C_18_H_22_NaO_3_]^+^ 309.1461).

2-(2-(Naphthalen-2-yl)ethoxy)acetic acid (**3d**). To a stirred solution of *tert*-butyl ester **10** (1 mmol) in dry CH_2_Cl_2_ (2 mL), under an inert argon atmosphere, trifluoroacetic acid (2 mL) was added and the reaction mixture was left stirring for 2 h. Toluene (2 mL) was then added and the solvents were evaporated under reduced pressure. The latter was repeated until complete removal of trifluoroacetic acid. The residue was diluted in H_2_O (5 mL) and diethyl ether (5 mL) and transferred to a separating funnel. An aqueous solution of 5% NaHCO_3_ (5 mL) was added, and the aqueous layer was acidified with an aqueous solution of 5% citric acid (5 mL) and then extracted with diethyl ether (3 × 5 mL). Concentration of the combined organic layers under reduced pressure afforded the desired product. Yield 81%; White solid; mp: 63–67 °C; ^1^H NMR (200 MHz, CD_3_OD): *δ* = 7.67−7.19 (m, 7H, 7 × ArH), 5.43 (br s, 1H, COOH), 4.02−3.88 (m, 2H, OCH_2_), 3.71−3.56 (m, 2H, OCH_2_), 2.97−2.85 (m, 2H, CH_2_Ar); ^13^C NMR (50 MHz, CD_3_OD): *δ* = 174.1, 137.3, 134.9, 133.5, 128.8, 128.4, 128.1, 126.8, 126.2, 73.1, 68.6, 36.9. HRMS (ESI) [M-H]^-^ *m/z*: 229.0866; (calculated for [C_14_H_13_O_3_]^-^ 229.0870).

1-(Benzo[*d*]thiazol-2-yl)-5-(naphthalen-2-yl)pentan-1-ol (**12**). To a stirred solution of benzothiazole (1.2 mmol) in dry diethyl ether (6.5 mL), at −78 °C, under an inert argon atmosphere, a solution of *n*-BuLi 1M (1.2 mmol) was added dropwise and the reaction mixture was stirred for 1h at −78 °C. A solution of aldehyde **11** (1 mmol) in dry diethyl ether (1.5 mL) was then added, and the reaction mixture was further stirred for 1 h at −78 °C and for 16 h at room temperature. The reaction was quenched with a saturated NH_4_Cl aqueous solution (5 mL), and the aqueous layer was collected and extracted with diethyl ether (2 x 10 mL). The combined organic layers were washed with brine (10 mL), dried over Na_2_SO_4_, and concentrated under reduced pressure. Purification by flash chromatography eluting with a mixture of EtOAc:petroleum ether (40–60 °C) 3:7 afforded the desired product. Yield 55%; Orange solid of low melting point; ^1^H NMR (400 MHz, CDCl_3_): *δ* = 7.98 (d, *J* = 8.1 Hz, 1H, ArH), 7.91−7.70 (m, 4H, 4 × ArH), 7.59 (s, 1H, ArH), 7.50−7.34 (m, 4H, ArH), 7.30 (d, *J* = 8.4 Hz, 1H, ArH), 5.10 (dd, *J_1_* = 7.5, *J_2_* = 4.7 Hz, 1H, C*H*OH), 3.83 (br s, 1H, OH), 2.77 (t, *J* = 7.6 Hz, 2H, CH_2_Ar), 2.14−1.93 (m, 2H, CH_2_), 1.84−1.70 (m, 2H, CH_2_), 1.70−1.50 (m, 2H, CH_2_); ^13^C NMR (100 MHz, CDCl_3_): *δ* = 176.8, 152.8, 140.0, 134.8, 133.7, 132.0, 127.9, 127.7, 127.5, 127.4, 126.4, 126.2, 125.9, 125.12, 125.09, 122.9, 121.9, 72.2, 38.0, 35.9, 31.1, 25.0; HRMS (ESI) [M+H]^+^ *m/z*: 348.1425; (calculated for [C_22_H_22_NOS]^+^ 348.1417).

General procedure for the synthesis of cyanohydrins **14a,b** and **15**.

To a stirred solution of aldehydes **13a,b**, **11** (1 mmol) in CH_2_Cl_2_ (1.3 mL), a solution of NaHSO_3_ (1.5 mmol, 156 mg) in water (0.3 mL) was added at room temperature. After stirring for 30 min, the organic solvent was concentrated under reduced pressure, water (0.3 mL) was added, and the reaction mixture was cooled to 0 °C. Then, a solution of KCN (1.5 mmol, 98 mg) in water (0.3 mL) was added dropwise over 1 h, and the reaction mixture was left stirring for 16 h. After the completion of the reaction, CH_2_Cl_2_ (5 mL) was added to extract the product and the organic layer was washed with brine (10 mL), dried over Na_2_SO_4_ and concentrated under reduced pressure. Purification by flash chromatography eluting with the appropriate mixture of EtOAc:petroleum ether (40–60 °C) afforded the desired product.

2-Hydroxy-6-(4-methoxyphenyl)hexanenitrile (**14a**). Yield 78%; Colorless oil; ^1^H NMR (200 MHz, CDCl_3_): *δ* = 7.16 (d, *J* = 8.0 Hz, 2H, 2 × ArH), 6.90 (d, *J* = 8.0 Hz, 2H, 2 × ArH), 4.45 (q, *J* = 6.0 Hz, 1H, C*H*OH), 4.11 (br s, 1H, OH), 3.82 (s, 3H, OCH_3_), 2.62 (t, *J* = 6.0 Hz, 2H, CH_2_Ar), 1.93−1.83 (m, 2H, CH_2_), 1.72−1.55 (m, 4H, 2 × CH_2_); ^13^C NMR (50 MHz, CDCl_3_): *δ* = 157.2, 133.9, 128.9, 119.9, 113.4, 60.6, 54.9, 34.5, 34.3, 30.6, 23.8; MS (ESI) *m/z* (%): 237.2 [(M+NH_4_)^+^, 100].

2-Hydroxy-3-(4-methoxyphenethoxy)propanenitrile (**14b**). Yield 40%; Colorless oil; ^1^H NMR (200 MHz, CDCl_3_): *δ* = 7.14 (d, *J* = 8.6 Hz, 2H, 2 × ArH), 6.86 (d, *J* = 8.8 Hz, 2H, 2 × ArH), 4.56−4.45 (m, 1H, C*H*OH), 3.79 (s, 3H, OCH_3_), 3.77−3.65 (m, 4H, 2 × CH_2_), 2.86 (t, *J* = 6.9 Hz, 2H, CH_2_); HRMS (ESI) [M+Na]^+^ *m/z*: 244.0944; (calculated for [C_12_H_15_NNaO_3_]^+^ 244.0944).

2-Hydroxy-6-(naphthalen-2-yl)hexanenitrile (**15**). Yield 45%; Colorless oil; ^1^H NMR (200 MHz, CDCl_3_): *δ* = 7.93−7.28 (m, 7H, 7 × ArH), 4.39 (q, *J* = 6.2 Hz, 1H, C*H*OH), 3.41 (br s, 1H, OH), 2.81 (t, *J* = 7.4 Hz, 2H, CH_2_Ar), 1.82−1.68 (m, 4H, 2 × CH_2_), 1.59−1.55 (m, 2H, CH_2_); ^13^C NMR (50 MHz, CDCl_3_): *δ* = 139.4, 133.4, 131.8, 127.8, 127.5, 127.3, 127.1, 126.3, 125.9, 125.1, 120.0, 60.9, 35.6, 34.8, 30.4, 24.1; HRMS (ESI) [M+Na]^+^ *m/z*: 262.1202; (calculated for [C_16_H_17_NNaO]^+^ 262.1202).

General procedure for the synthesis of α-hydroxy benzoxazoles (**16a,c,d**) and α-hydroxy benzimidazoles (**16b,e**) from cyanohydrins.

To a stirred mixture of chloroform (0.5 M) and absolute ethanol (0.5 M) cooled at 0 °C, under an inert dry argon atmosphere, acetyl chloride (0.46 mL) was added dropwise over 15 min. Then, a solution of cyanohydrins **14a,b** and **15** (1 mmol) in CHCl_3_ (0.5 M) was added and the reaction mixture was stirred at 0 °C for 1 h. The solvent was evaporated under reduced pressure and at a temperature not higher than 25 °C. The reaction mixture was then dissolved in absolute ethanol (1.2 M), 2-aminophenol (for the benzoxazole compounds **16a,c,d**) or 2-phenylenediamine (for the benzimidazole compounds **16b,e**) (1.1 mmol) was added and the final reaction mixture was refluxed under an inert argon atmosphere for 16 h. After completion of the reaction, the solvent was evaporated under reduced pressure. Purification by flash chromatography eluting with the appropriate mixture of EtOAc:petroleum ether (40–60 °C) afforded the desired product.

1-(Benzo[*d*]oxazol-2-yl)-5-(4-methoxyphenyl)pentan-1-ol (**16a**). Yield 57%; Pale yellow oil; ^1^H NMR (200 MHz, CDCl_3_): *δ* = 7.73−7.67 (m, 1H, ArH), 7.57−7.48 (m, 1H, ArH), 7.39−7.30 (m, 2H, 2 × ArH), 7.08 (d, *J* = 8.0 Hz, 2H, 2 × ArH), 6.81 (d, *J* = 8.0 Hz, 2H, 2 × ArH), 4.97 (t, *J* = 6.0 Hz, 1H, C*H*OH), 3.84 (br s, 1H, OH), 3.78 (s, 3H, OCH_3_), 2.57 (t, *J* = 6.0 Hz, 2H, CH_2_Ar), 2.15–1.94 (m, 2H, CH_2_), 1.73−1.47 (m, 4H, 2 × CH_2_); ^13^C NMR (50 MHz, CDCl_3_): *δ* = 167.8, 157.5, 150.6, 140.2, 134.3, 129.1, 125.1, 124.4, 119.8, 113.6, 110.7, 67.9, 55.1, 35.3, 34.7, 31.3, 24.5; MS (ESI) *m/z* (%): 312.2 [(M+H)^+^, 100].

1-(1*H*-Benzo[*d*]imidazol-2-yl)-5-(4-methoxyphenyl)pentan-1-ol (**16b**). Yield 37%; White solid; mp: 165–167 °C; ^1^H NMR (200 MHz, CD_3_OD): *δ* = 7.58−7.54 (m, 2H, 2 × ArH), 7.25−7.21 (m, 2H, 2 × ArH), 7.03 (d, *J* = 8.0 Hz, 2H, 2 × ArH), 6.75 (d, *J* = 8.0 Hz, 2H, 2 × ArH), 4.93 (t, *J* = 6.0 Hz, 1H, C*H*OH), 3.73 (s, 3H, OCH_3_), 2.54 (t, *J* = 6.0 Hz, 2H, CH_2_Ar), 2.05−1.91 (m, 2H, CH_2_), 1.66−1.40 (m, 4H, 2 × CH_2_); ^13^C NMR (50 MHz, CD_3_OD): *δ* = 159.3, 139.4, 135.8, 135.7, 130.4, 123.4, 115.8, 114.7, 69.5, 55.7, 37.8, 35.8, 32.7, 25.7; MS (ESI) *m/z* (%): 311.2 [(M+H)^+^, 100].

1-(Benzo[*d*]oxazol-2-yl)-2-(4-methoxyphenethoxy)ethan-1-ol (**16c**). Yield 65%; Pale yellow solid of low melting point; ^1^H NMR (200 MHz, CDCl_3_): *δ* = 7.80−7.69 (m, 1H, ArH), 7.56−7.47 (m, 1H, ArH), 7.38−7.30 (m, 2H, 2 × ArH), 7.03 (d, *J* = 8.5 Hz, 2H, 2 × ArH), 6.71 (d, *J* = 8.6 Hz, 2H, 2 × ArH), 5.12 (t, *J* = 4.9 Hz, 1H, C*H*OH), 4.01−3.91 (m, 2H, OC*H_2_*CH), 3.81−3.59 (m, 5H, OCH_3_, OCH_2_), 2.79 (t, *J* = 6.9 Hz, 2H, CH_2_); ^13^C NMR (50 MHz, CDCl_3_): *δ* = 165.0, 158.0, 157.6, 140.5, 130.5, 129.7, 125.2, 124.5, 120.1, 113.7, 110.8, 72.7, 72.5, 67.5, 55.1, 35.0; HRMS (ESI) [M+Na]^+^ *m/z*: 336.1201; (calculated for [C_18_H_19_NNaO_4_]^+^ 336.1206).

1-(Benzo[*d*]oxazol-2-yl)-5-(naphthalen-2-yl)pentan-1-ol (**16d**). Yield 53%; Orange oil; ^1^H NMR (200 MHz, CDCl_3_): *δ* = 7.94–7.20 (m, 11H, 11 × ArH, 5.08–4.93 (m, 1H, C*H*OH), 4.45 (br s, 1H, OH), 2.79 (t, *J* = 7.4 Hz, 2H, CH_2_Ar), 2.10 (m, 2H, CH_2_), 1.89−1.49 (m, 4H, 2 × CH_2_); ^13^C NMR (50 MHz, CDCl_3_): *δ* = 168.0, 150.5, 140.2, 139.8, 133.5, 131.8, 127.7, 127.5, 127.3, 127.2, 126.2, 125.8, 125.1, 125.0, 124.4, 119.8, 110.7, 67.8, 35.8, 35.2, 30.9, 24.7; HRMS (ESI) [M+Na]^+^ *m/z*: 354.1466; (calculated for [C_22_H_21_NNaO_2_]^+^ 354.1465).

1-(1*H*-Benzo[*d*]imidazol-2-yl)-5-(naphthalen-2-yl)pentan-1-ol (**16e**). Yield 43%; White solid; ^1^H NMR (200 MHz, CDCl_3_): *δ* = 7.82−7.03 (m, 11H, 11 × ArH), 4.85 (t, *J* = 6.6 Hz, 1H, C*H*OH), 4.41 (br s, 1H, OH), 2.65 (t, *J* = 7.3 Hz, 2H, CH_2_Ar), 2.03−1.76 (m, 2H, CH_2_), 1.74−1.25 (m, 4H, 2 × CH_2_); ^13^C NMR (50 MHz, CDCl_3_): *δ* = 157.4, 139.8, 137.6, 133.4, 131.7, 127.5, 127.4, 127.2, 127.1, 126.1, 125.6, 124.8, 122.3, 114.7, 68.1, 36.3, 35.7, 30.9, 24.7; HRMS (ESI) [M+H]^+^ *m/z*: 331.1805; (calculated for [C_22_H_23_N_2_O]^+^ 331.1805).

General procedure for the synthesis of *O*-acyl-amidoximes (**20b,c**).

To a stirred solution of amidoxime **19** (1.0 mmol) in dry CH_2_Cl_2_ (20 mL), benzoic acid (for benzoate group) or isobutyric anhydride (for isobutyrate group) (1 mmol, 102 mg) and *N*,*N′*-dicyclohexylcarbodiimide (DCC) (1.1 mmol, 227 mg) were added. The reaction mixture was stirred for 24 h at room temperature. After completion of the reaction the organic solvent was evaporated under reduced pressure. Purification by flash chromatography eluting with the appropriate mixture of EtOAc:petroleum ether (40–60 °C) afforded the desired product.

(*Z*)-*N’*-(Benzoyloxy)-2-((*tert*-butyldimethylsilyl)oxy)-6-(4-methoxyphenyl)hexanimidamide (**20b**). Yield 67%; ^1^H NMR (200 MHz, CDCl_3_): *δ* = 8.08−7.98 (m, 2H, 2 × ArH), 7.60−7.50 (m, 1H, ArH), 7.48−7.37 (m, 2H, 2 × ArH), 7.07 (d, *J* = 8.6 Hz, 2H, 2 × ArH), 6.79 (d, *J* = 8.6 Hz, 2H, 2 × ArH), 5.07 (s, 2H, NH_2_), 4.41 (t, *J* = 6.2 Hz, 1H, OCH), 3.75 (s, 3H, OCH_3_), 2.55 (t, *J* = 7.2 Hz, 2H, CH_2_Ar), 1.66−1.42 (m, 4H, 2 × CH_2_), 1.32−1.08 (m, 2H, CH_2_), 0.90 (s, 9H, 3 × CCH_3_), 0.09 (s, 6H, 2 × SiCH_3_); ^13^C NMR (50 MHz, CDCl_3_): *δ* = 164.2, 161.0, 157.8, 134.7, 133.1, 129.9, 129.6, 129.5, 128.6, 113.9, 70.5, 55.4, 37.8, 35.0, 31.7, 25.9, 24.9, 18.3, -4.9; HRMS (ESI) [M+H]^+^ *m/z*: 471.2670; (calculated for [C_26_H_39_N_2_O_4_Si]^+^ 471.2674).

(*Z*)-2-((*tert*-Butyldimethylsilyl)oxy)-*N’*-(isobutyryloxy)-6-(4-methoxyphenyl)hexanimidamide (**20c**). Yield 95%; ^1^H NMR (200 MHz, CDCl_3_): *δ* = 7.05 (d, *J* = 8.5 Hz, 2H, 2 × ArH), 6.79 (d, *J* = 8.6 Hz, 2H, 2 × ArH), 4.92 (s, 2H, NH_2_), 4.30 (t, *J* = 6.2 Hz, 1H, OCH), 3.76 (s, 3H, OCH_3_), [2.77−2.57 m, 1H, C*H*(CH_3_)_2_], 2.52 (t, *J* = 7.3 Hz, 2H, CH_2_Ar), 1.78−1.33 (m, 6H, 3 × CH_2_), 1.22 (d, *J* = 7.0 Hz, 6H, 2 × CHC*H_3_*), 0.87 (s, 9H, 3 × CCH_3_), 0.05 (s, 6H, 2 × SiCH_3_); ^13^C NMR (50 MHz, CDCl_3_): *δ* = 174.1, 160.1, 157.6, 134.6, 129.3, 113.7, 70.2, 55.2, 37.5, 34.8, 33.2, 31.5, 25.7, 24.7, 19.3, 18.0, -5.1; HRMS (ESI) [M+H]^+^ *m/z*: 437.2826; (calculated for [C_23_H_41_N_2_O_4_Si]^+^ 437.2830).

General procedure for the synthesis of *α*-hydroxy-oxadiazoles (**21b,c**).

To a stirred solution of *O*-acyl-amidoximes **20b,c** (1.0 mmol) in dry toluene (3 mL) in a microwave vessel, tetrabutylammonium fluoride (TBAF) (1 M in THF, 1.0 mmol) was added. The reaction mixture was left stirring under microwave irradiation (initial setting at 90 W) for 1 h at 120 °C. The organic solvent was evaporated under reduced pressure. Purification by flash chromatography eluting with the appropriate mixture of EtOAc:petroleum ether (40–60 °C) afforded the desired product.

5-(4-Methoxyphenyl)-1-(5-phenyl-1,2,4-oxadiazol-3-yl)pentan-1-ol (**21b**). Yield 38%; White solid; mp: 88–90 °C; ^1^H NMR (200 MHz, CDCl_3_): *δ* = 8.26−8.05 (m, 2H, 2 × ArH), 7.67−7.46 (m, 3H, 3 × ArH), 7.08 (d, *J* = 8.6 Hz, 2H, 2 × ArH), 6.80 (d, *J* = 8.6 Hz, 2H, 2 × ArH), 4.93 (t, *J* = 6.6 Hz, 1H, C*H*OH), 3.76 (s, 3H, OCH_3_), 2.97 (br s, 1H, OH), 2.57 (t, *J* = 7.3 Hz, 2H, CH_2_Ar), 2.12−1.91 (m, 2H, CH_2_), 1.78−1.40 (m, 4H, 2 × CH_2_); ^13^C NMR (50 MHz, CDCl_3_): *δ* = 176.0, 172.8, 157.7, 134.6, 133.0, 129.3, 129.2, 128.3, 124.1, 113.8, 66.8, 55.3, 35.6, 34.9, 31.5, 24.8; HRMS (ESI) [M+H]^+^ *m/z*: 339.1700; (calculated for [C_20_H_23_N_2_O_3_]^+^ 339.1703).

1-(5-Isopropyl-1,2,4-oxadiazol-3-yl)-5-(4-methoxyphenyl)pentan-1-ol (**21c**). Yield 51%; Pale yellow oil; ^1^H NMR (200 MHz, CDCl_3_): *δ* = 7.05 (d, *J* = 8.7 Hz, 2H, 2 × ArH), 6.79 (d, *J* = 8.7 Hz, 2H, 2 × ArH), 4.80 (t, *J* = 6.6 Hz, 1H, C*H*OH), 3.75 (s, 3H, OCH_3_), 3.30−3.08 [m, 1H, C*H*(CH_3_)_2_], 2.83 (br s, 1H, OH), 2.54 (t, *J* = 7.4 Hz, 2H, CH_2_Ar), 1.98−1.83 (m, 2H, CH_2_), 1.75−1.47 (m, 4H, 2 × CH_2_), 1.37 (d, *J* = 7.0 Hz, 6H, 2 × CHC*H_3_*); ^13^C NMR (50 MHz, CDCl_3_): *δ* = 184.3, 171.9, 157.7, 134.6, 129.3, 113.8, 66.7, 55.3, 35.5, 34.9, 31.4, 27.6, 24.8, 20.2; HRMS (ESI) [M+H]^+^ *m/z*: 305.1858; (calculated for [C_17_H_25_N_2_O_3_]^+^ 305.1860).

General procedure for the oxidation of secondary alcohols to ketones (**17a**–**e**, **22b,c**).

To a stirred solution of α-hydroxy-heterocyclic compounds **16a**–**e** and **21b,c** (1 mmol) in dry CH_2_Cl_2_ (0.2 M), under an inert argon atmosphere, Dess–Martin periodinane was added (1.3 mmol, 551 mg). The reaction mixture was stirred for 1 h and after completion of the reaction the solvent was evaporated under reduced pressure and Et_2_O (30 mL) was added. The organic phase was washed with saturated aqueous NaHCO_3_ (20 mL) containing Na_2_S_2_O_3_ (1.5 g, 9.5 mmol), H_2_O (20 mL), dried over Na_2_SO_4_, and the organic solvent was evaporated under reduced pressure. Purification by flash chromatography eluting with the appropriate mixture of EtOAc:petroleum ether (40–60 °C) afforded the desired product.

1-(Benzo[*d*]oxazol-2-yl)-5-(4-methoxyphenyl)pentan-1-one (**17a**). Yield 88%; White solid; mp: 59–61 °C; ^1^H NMR (200 MHz, CDCl_3_): *δ* = 7.90 (d, *J* = 7.4 Hz, 1H, ArH), 7.66 (d, *J* = 7.6 Hz, 1H, ArH), 7.59–7.38 (m, 2H, 2 × ArH), 7.11 (d, *J* = 8.5 Hz, 2H, 2 × ArH), 6.82 (d, *J* = 8.5 Hz, 2H, 2 × ArH), 3.77 (s, 3H, OCH_3_), 3.24 (t, *J* = 7.1 Hz, 2H, CH_2_CO), 2.63 (t, *J* = 7.3 Hz, 2H, CH_2_Ar), 1.95–1.60 (m, 4H, 2 × CH_2_); ^13^C NMR (50 MHz, CDCl_3_): *δ* = 190.2, 157.7, 157.2, 150.7, 140.5, 134.1, 129.3, 128.5, 125.8, 122.3, 113.7, 112.0, 55.3, 39.4, 34.7, 31.1, 23.4; HRMS (ESI) [M-H]^-^ *m/z*: 308.1291; (calculated for [C_19_H_18_NO_3_]^-^ 308.1292).

1-(1*H*-Benzo[*d*]imidazol-2-yl)-5-(4-methoxyphenyl)pentan-1-one (**17b**). Yield 79%; Colorless solid; mp: 101–103 °C; ^1^H NMR (200 MHz, CDCl_3_): *δ* = 10.64 (br s, 1H, NH), 8.00−7.82 (m, 1H, ArH), 7.63−7.31 (m, 3H, 3 × ArH), 7.10 (d, *J* = 8.4 Hz, 2H, 2 × ArH), 6.81 (d, *J* = 8.6 Hz, 2H, 2 × ArH), 3.77 (s, 3H), 3.34 (t, *J* = 7.2 Hz, 2H, CH_2_CO), 2.62 (t, *J* = 7.3 Hz, 2H, CH_2_Ar), 1.96−1.62 (m, 4H, 2 × CH_2_); ^13^C NMR (50 MHz, CDCl_3_): *δ* = 194.7, 157.8, 151.1, 147.6, 134.3, 129.4, 126.6, 124.0, 122.0, 114.1, 113.8, 112.3, 55.4, 38.2, 34.9, 31.3, 23.7; HRMS (ESI) [M-H]^−^ *m/z*: 307.1455; (calculated for [C_19_H_19_N_2_O_2_]^-^ 308.1292).

1-(Benzo[*d*]oxazol-2-yl)-2-(4-methoxyphenethoxy)ethan-1-one (**17c**). Yield 35%; Pale yellow solid of low melting point; ^1^H NMR (500 MHz, CDCl_3_): *δ* = 7.87 (d, *J* = 8.0 Hz, 1H, ArH), 7.66 (d, *J* = 8.2 Hz, 1H, ArH), 7.55 (t, *J* = 7.8 Hz, 1H, ArH), 7.47 (t, *J* = 7.8 Hz, 1H, ArH), 7.18 (d, *J* = 8.5 Hz, 2H, 2 × ArH), 6.83 (d, *J* = 8.3 Hz, 2H, 2 × ArH), 5.01 (s, 2H, OCH_2_CO), 3.84 (t, *J* = 7.0 Hz, 2H, OCH_2_), 3.77 (s, 3H, OCH_3_), 2.97 (t, *J* = 7.0 Hz, 2H, CH_2_Ar); ^13^C NMR (125 MHz, CDCl_3_): *δ* = 186.4, 158.3, 155.5, 150.6, 140.4, 130.4, 130.0, 128.9, 126.1, 122.4, 114.0, 112.1, 73.8, 73.4, 55.4, 35.4; IR: $\tilde{v}$ = 3091, 1718, 1612, 1515, 1453 cm^-1^; HRMS (ESI) [M+H]^+^ *m/z*: 312.1230; (calculated for [C_18_H_18_NO_4_]^+^ 312.1230); HRMS (ESI) [M+Na]^+^ *m/z*: 334.1051; (calculated for [C_18_H_17_NNaO_4_]^+^ 334.1050).

1-(Benzo[*d*]oxazol-2-yl)-5-(naphthalen-2-yl)pentan-1-one (**17d**). Yield 96%; White solid; 77−82 °C; ^1^H NMR (400 MHz, CDCl_3_): *δ* = 7.89 (d, *J* = 8.0 Hz, 1H, ArH), 7.84–7.72 (m, 3H, 3 × ArH), 7.69−7.60 (m, 2H, 2 × ArH), 7.56−7.38 (m, 4H, 4 × ArH), 7.34 (dd, *J_1_* = 8.4, *J_2_* = 1.4 Hz, 1H, ArH), 3.27 (t, *J* = 7.0 Hz, 2H, CH_2_CO), 2.86 (t, *J* = 7.2 Hz, 2H, CH_2_Ar), 1.98−1.79 (m, 4H, 2 × CH_2_); ^13^C NMR (100 MHz, CDCl_3_): *δ* = 190.1, 157.3, 150.8, 140.6, 139.6, 133.7, 132.1, 128.6, 128.0, 127.7, 127.5, 127.3, 126.5, 126.0, 125.8, 125.2, 122.3, 112.0, 39.4, 35.8, 30.7, 23.6; IR: $\tilde{v}$ = 3049, 1701, 1601, 1534, 1506 cm^-1^; HRMS (ESI) [M+H]^+^ *m/z*: 330.1499; (calculated for [C_22_H_20_NO_2_]^+^ 330.1489).

1-(1*H*-Benzo[*d*]imidazol-2-yl)-5-(naphthalen-2-yl)pentan-1-one (**17e**). Yield 45%; White solid; mp: 127−132 °C; ^1^H NMR (400 MHz, CDCl_3_): *δ* = 10.24 (s, 1H, NH), 7.91 (d, *J* = 8.0 Hz, 1H, ArH), 7.82–7.73 (m, 3H, 3 × ArH), 7.62 (s, 1H, ArH), 7.53 (d, *J* = 7.9 Hz, 1H, ArH), 7.46−7.31 (m, 5H, 5 × ArH), 3.35 (t, *J* = 6.9 Hz, 2H, CH_2_CO), 2.85 (t, *J* = 7.2 Hz, 2H, CH_2_Ar), 1.96−1.80 (m, 4H, 2 × CH_2_); ^13^C NMR (50 MHz, CDCl_3_): *δ* = 194.4, 147.6, 143.5, 139.8, 133.7, 133.5, 132.1, 128.0, 127.7, 127.5, 127.4, 126.63, 126.56, 126.0, 125.2, 124.0, 122.1, 112.2, 38.2, 35.9, 30.9, 23.7; IR: $\tilde{v}$ = 3286, 3055, 1679, 1598, 1512, 1401 cm^−1^; HRMS (ESI) [M+H]^+^ *m/z*: 329.1655; (calculated for [C_22_H_21_N_2_O]^+^ 329.1648).

5-(4-Methoxyphenyl)-1-(5-phenyl-1,2,4-oxadiazol-3-yl)pentan-1-one (**22b**). Yield 77%; White solid; 85−87 °C; ^1^H NMR (200 MHz, CDCl_3_): *δ* = 8.30−8.13 (m, 2H, 2 × ArH), 7.72−7.46 (m, 3H, 3 × ArH), 7.10 (d, *J* = 8.6 Hz, 2H, 2 × ArH), 6.82 (d, *J* = 8.7 Hz, 2H, 2 × ArH), 3.78 (s, 3H, OCH_3_), 3.14 (t, *J* = 7.1 Hz, 2H, CH_2_CO), 2.62 (t, *J* = 7.3 Hz, 2H, CH_2_Ar), 1.93–1.62 (m, 4H, 2 × CH_2_); ^13^C NMR (50 MHz, CDCl_3_): *δ* = 191.8, 177.2, 166.2, 157.9, 134.1, 133.6, 129.4, 128.6, 123.5, 113.9, 55.3, 40.7, 34.8, 31.1, 23.2; HRMS (ESI) [M+Na]^+^ *m/z*: 359.1363; (calculated for [C_20_H_20_N_2_NaO_3_]^+^ 359.1366).

1-(5-Isopropyl-1,2,4-oxadiazol-3-yl)-5-(4-methoxyphenyl)pentan-1-one (**22c**). Yield 45%; Colorless oil; ^1^H NMR (200 MHz, CDCl_3_): *δ* = 7.09 (d, *J* = 8.4 Hz, 2H, 2 × ArH), 6.81 (d, *J* = 8.4 Hz, 2H, 2 × ArH), 3.78 (s, 3H, OCH_3_), 3.38−3.21 [m, 1H, C*H*(CH_3_)_2_], 3.07 (t, *J* = 7.0 Hz, 2H, CH_2_CO), 2.59 (t, *J* = 7.2 Hz, 2H, CH_2_Ar), 1.84–1.61 (m, 4H, 2 × CH_2_), 1.44 (d, *J* = 7.0 Hz, 6H, 2 × CHC*H_3_*); ^13^C NMR (50 MHz, CDCl_3_): *δ* = 191.9, 185.7, 165.6, 157.9, 134.2, 129.4, 113.9, 55.4, 40.5, 34.8, 31.1, 27.7, 23.2, 20.2; HRMS (ESI) [M+Na]^+^ *m/z*: 325.1521; (calculated for [C_17_H_22_N_2_NaO_3_]^+^ 325.1523).

### 2.2. In Vitro Suppression of Cytokine-Triggered PGE_2_ Generation in Renal Mesangial Cells

#### 2.2.1. Cell Culture

Rat renal mesangial cells (clone MZ B1) were isolated and characterized as previously described [22] and cultivated in medium consisting of RPMI 1640 supplemented with 10% fetal bovine serum, 10 mM Hepes, pH 7.4, 6 µg/mL bovine insulin, 5 mg/mL transferrin, 5 nM sodium selenite, 100 units/mL penicillin, and 100 µg/mL streptomycin. Prior to stimulation, cells were incubated for 4 h in DMEM containing 10 mM Hepes, pH 7.4, and 0.1 mg/mL fatty acid-free bovine serum albumin (BSA).

#### 2.2.2. Quantification of Prostagladin E_2_

Confluent mesangial cells in 24-well plates were stimulated for 24 h in a total volume of 400 µL DMEM containing 0.1 mg/mL BSA with the stimuli and inhibitors as indicated in the figure legends. Thereafter, supernatants were removed and centrifuged for 5 min at 1000 x g. The supernatant was taken for PGE_2_ quantification using an enzyme-linked immunoassay (Enzo Life Sciences, Lörrach, Germany) following exactly the manufacturer’s recommendations.

#### 2.2.3. Statistical Analysis

Statistical analysis of data was performed using one-way analysis of variance (ANOVA) followed by a Bonferroni’s post hoc test for multiple comparisons (GraphPad Prism version 5.00, San Diego, CA, USA). Half-maximal effective concentrations (EC_50_) of the compounds were calculated using the same software.

## 3. Results and Discussion

### 3.1. Synthesis of Inhibitors

**Figure 3:**
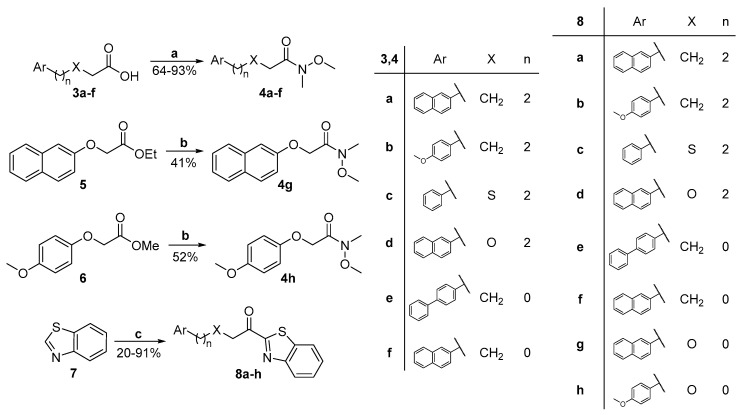
Synthesis of α-ketobenzothiazoles **8a**–**h**. (**a**) HN(OMe)Me·HCl, WSCI·HCl, DMAP, NMM, CH_2_Cl_2_; (**b**) (i) HN(OMe)Me·HCl, dry THF, −20 °C; (ii) *i*-PrMgCl, -20 °C; and (**c**) (i) *n*-BuLi, dry Et_2_O, −78 °C; (ii) Weinreb amides **4a**–**h**, dry Et_2_O, −78 °C to rt.

Benzothiazolyl ketones were synthesized in two steps, most of them starting from the corresponding carboxylic acids, as shown in Figure 3. The first step involves the formation of the Weinreb amides **4a**–**f** by coupling of carboxylic acids **3a**–**f** with *N*,*O*-dimethylhydroxylamine hydrochloride using *N*-(3-dimethylaminopropyl)-*N’*-ethyl carbodiimide hydrochloride (WSCI·HCl) as the coupling agent [17,23]. Weinreb amides **4g,h** were prepared from esters **5** and **6** using *N*,*O*-dimethylhydroxylamine hydrochloride, in the presence of isopropylmagnesium chloride (*i*-PrMgCl) [24]. A nucleophilic attack of benzothiazolyl lithium to Weinerb amides **4a**–**h** afforded the desired α-heterocyclic ketones **8a**–**h** (Figure 3).

**Figure 4:**
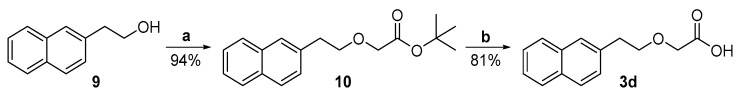
Synthesis of compound **3d**. (a) BrCH_2_COOC(CH_3_)_3_, Bu_4_NHSO_4_, 50% NaOH, toluene; (b) 50% TFA, dry CH_2_Cl_2_.

Carboxylic acid **3d,** required for the synthesis of **8d**, was prepared in two steps via etherification of alcohol **9** with *tert*-butyl 2-bromoacetate, followed by deprotection using trifluoroacetic acid (TFA) (Figure 4).

**Figure 5:**
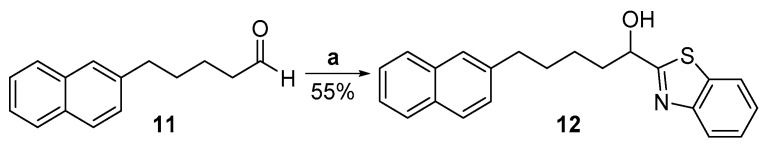
Synthesis of compound **12**. (a) Benzothiazole, *n*-BuLi, dry Et_2_O, −78 °C to rt.

α-Hydroxy-derivative **12** was synthesized through a reaction between aldehyde **11** and benzothiazolyl lithium (Figure 5) [25].

**Figure 6:**
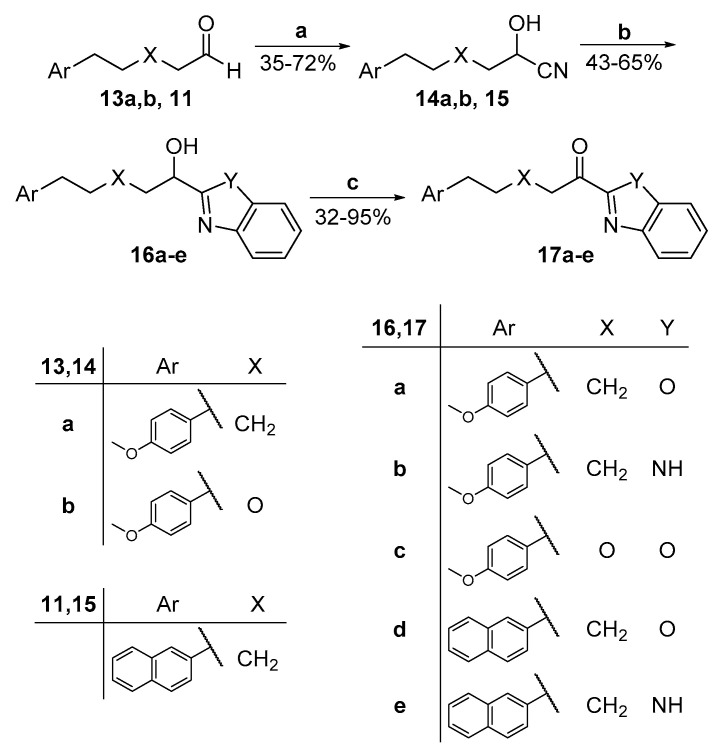
Synthesis of α-ketobenzoxazoles **17a,c,d** and α-ketobenzimidazoles **17b,e**. (a) (i) NaHSO_3_, CH_2_Cl_2_; (ii) KCN, H_2_O; (b) (i) CH_3_COCl, CHCl_3_/absolute EtOH; (ii) 2-aminophenol or 2-phenylenediamine, absolute EtOH; (c) Dess–Martin periodinane, CH_2_Cl_2_.

For the benzoxazoles and benzimidazoles **17a**–**e**, the synthesis started from the corresponding aldehydes **13a,b** and **11,** which were converted to the corresponding cyanohydrins **14a,b** and **15**. The formation of the heterocyclic rings was accomplished by the treatment of these compounds with 2-aminophenol (for the benzoxazole derivatives **16a,c,d**) or 2-phenylenediamine (for the benzimidazole derivatives **16b,e**) in the presence of acetyl chloride [26]. Hydroxy compounds **16a**–**e** were finally oxidized to the α-ketoheterocycles **17a**–**e** using Dess–Martin periodinane (Figure 6).

**Figure 7:**
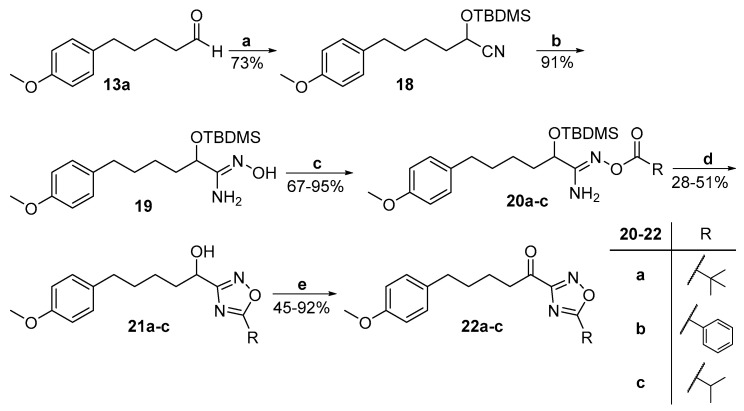
Synthesis of α-keto-1,2,4-oxadiazoles **22a**–**c**. (a) TBDMSCN, KCN, 18-crown-6, dry CH_2_Cl_2_; (b) 50% aq. NH_2_OH, microwave irradiation 50 W, 120 °C; (c) pivalic acid (for pivalate group) or benzoic acid (for benzoate group) or isobutyric anhydride (for isobutyrate group), DCC, dry CH_2_Cl_2_; (d) TBAF, toluene, microwave irradiation 90 W, 120 °C; (e) Dess–Martin periodinane, CH_2_Cl_2_.

The synthesis of keto-1,2,4-oxadiazoles was accomplished following a previously published procedure, as depicted in Figure 7. Amidoxime **19** was synthesized from aldehyde **13a** through the corresponding O-*tert*-bytyldimethylsilyl cyanide **18** [21]. It was then coupled with either pivalic acid [21], benzoic acid, or isobutyric anhydride, using *N*,*N′*-dicyclohexylcarbodiimide (DCC) as the coupling reagent to afford compounds **20a**–**c**. The cyclization of these *O*-acyl-amidoximes took place in the presence of tetrabutylammonium fluoride (TBAF) under microwave irradiation, giving the desired hydroxy-oxadiazole derivatives **21a**–**c**, which were then subjected to oxidation with Dess-Martin periodinane, providing the final α-keto-oxadiazoles **22a**–**c** (Figure 7).

In the ^1^H-NMR spectra of the final heterocyclic compounds, the most characteristic peaks are those corresponding to the protons of the aromatic fused ring, which are located closest to the heteroatoms. These protons are the most downfield shifted aromatic ones, appearing at 8.24−7.92 ppm in the case of α-ketobenzothiazoles **8a**–**h**, and at 7.90−7.31 in the case of α-ketobenzoxazoles **17a,c,d** and α-ketobenzimidazoles **17b,e**. In addition, the ^1^H-NMR spectra of α-ketobenzimidazoles **17b,e** show a characteristic chemical shift of N-H above 10 ppm. In the ^13^C-NMR spectra of α-ketobenzothiazoles **8a,b,e,f**, the carbon atom of the carbonyl group resonates at 195.5–194.4 ppm, while the presence of an oxygen or a sulfur atom at the β-position of the alkyl chain (compounds **8c,d,g,h**) causes an upfield shift to 191.2−188.7 ppm. A characteristic chemical shift for the carbonyl carbon atom at 190.2−186.4 ppm, 194.7−194.4 ppm, and 191.9-191.8 ppm, is observed in the ^13^C-NMR spectra of α-ketobenzoxazoles **17a,c,d**, α-ketobenzimidazoles **17b,e**, and **22b,c**, α-keto-1,2,4-oxadiazoles, respectively.

### 3.2. Study of the Suppression of PGE_2_ Generation in Mesangial Cells

Renal mesangial cells were chosen as a model to evaluate the ability of our synthetic compounds to suppress the production of PGE_2_, based on our previous studies on PLA_2_ inhibitors [15,16]. Mesangial cells located in the renal glomerulus are involved in various pathological processes, including inflammation, of the renal glomerulus. As shown by Huwiler et al. [27,28], different PLA_2_s operate in mesangial cells to initiate the generation of PGE_2_. Stimulation of rat renal mesangial cells by interleukin-1β (IL-1β) plus forskolin (Fκ) results in huge increase of PGE_2_ synthesis, as previously described [15,16,29]. All the synthetic compounds were tested at a concentration of 3 µM and the results are summarized in Table 1.

The α-ketobenzothiazolyl derivative GK181, where the naphthalene group was placed at a distance of four carbon atoms from the carbonyl group, exhibited 85% inhibition of PGE_2_ release at a concentration of 3 µM (entry 1). When the distance between the naphthalene and the heterocyclic group was reduced to two carbon atoms (GK517, entry 2), the activity was abolished. Similarly, GK489 (entry 3), where an oxygen atom was introduced at the β-carbon atom to the carbonyl did not present any inhibitory activity. The reduction of the carbonyl group of GK181 to the corresponding alcohol (GK490, entry 4) also destroyed the inhibitory activity, highlighting the importance of the carbonyl group. Replacement of the benzothiazolyl group of GK181 by a benzoxazolyl one (GK491, entry 5) led led to a slight decrease of the activity (77%) in comparison to GK181, while the corresponding benzimidazolyl derivative (GK492, entry 6) exhibited even lower activity (57%).

Then, the naphthalene group was replaced by a p-methoxy-phenyl group. Compound GK299 (entry 7) exhibited a slightly decreased inhibitory activity (79%) in comparison to GK181 (entry 1). Keeping constant the p-methoxy-phenyl group and its distance from the carbonyl (four carbon atoms), the effect of various heterocyclic rings was examined. In all cases (GK355, GK358, GK367 GK368, and GK369, entries 8-12, respectively), the inhibitory potency was either reduced or diminished. Only compounds GK355 and GK368 (entries 8 and 11, respectively) containing a benzoxazolyl or a phenyl substituted oxadiazolyl ring, respectively, presented inhibitory activity (25% and 68%, respectively). Compound GK358 (entry 9), containing a benzimidazolyl group; GK367 (entry 10); and GK369 (entry 12), containing either a *tert*-butyl or an isopropyl substituted oxadiazolyl ring, did not present any inhibitory activity. Taking into account the results obtained for the various heterocyclic systems, either for the naphthalene-containing compounds or for the p-methoxy-phenyl-containing compounds, it seems that the benzothiazolyl group is the optimum heterocyclic system. The benzoxazole derivative GK453 (entry 13), as well as the benzothiazole derivatives GK455 (entry 14), GK516 (entry 15), GK518 (entry 16), and GK519 (entry 17) were proven unable to cause any inhibition.

**Figure 8:**
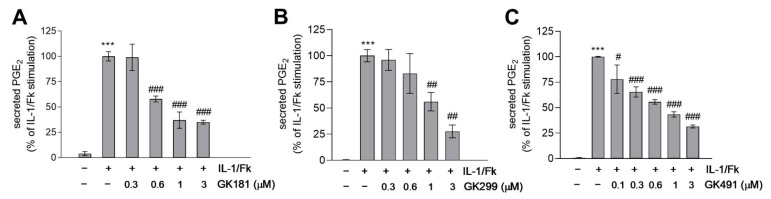
Effect of compounds (**A**) GK181, (**B**) GK299, and (**C**) GK491 on IL-1/Fk-stimulated PGE_2_ formation in mesangial cells. Cells were pretreated for 20 min with the indicated concentrations of GK compounds and then stimulated for 24 h in the absence (−) or presence (+) of 1 nM interleukin 1β(IL-1) plus 5 µM forskolin (Fk). Supernatants were taken for PGE_2_ quantification using an enzyme-linked immunoassay as described in the Methods section. Data are presented as % of maximal IL-1/Fk stimulation and are means S.D. (*n* = 3). *** *p* < 0.001 considered statistically significant when compared to the unstimulated samples; ^#^ *p* < 0.05, ^##^ *p* < 0.01, ^###^ *p* < 0.001 compared to the IL-1/Fk-stimulated samples.

The activity of compounds GK181, GK299, and GK491, which exhibited the highest potency at 3 µM, was further explored at various concentrations and the results are shown in Figure 8. GK181, GK299, and GK491 compounds presented potent inhibition of PGE_2_ generation with EC_50_ values of 0.71 µM, 1.42 µM, and 0.79 µM, respectively. In conclusion, we have identified one α-ketobenzothiazolyl derivative (GK181) and one α-ketobenzoxazolyl derivative (GK491), being able to inhibit the generation of PGE_2_ in renal mesangial cells at a nanomolar level.

### 3.3. Docking Studies

We have previously shown that inhibition of secreted PLA_2_ is a possible path via which synthetic compounds may evoke the suppression of PGE_2_ release [15,16]. Thus, we performed docking calculations to understand how these compounds may interact with the active site of secreted GIIA sPLA_2_. AutoDock Vina [30] was used for docking the most potent compounds GK181 and GK491 in GIIA sPLA_2_. The crystal structure of the enzyme was retrieved from the Brookhaven Protein Databank (PDB: 1KQU).

**Figure 9:**
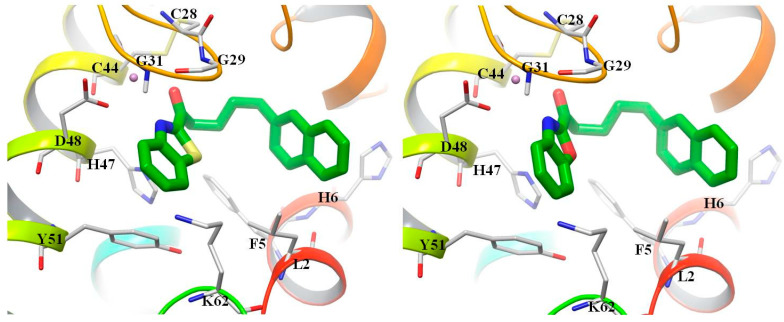
Proposed binding modes of GK181 (left) and GK491 (right) in the active site of GIIA sPLA_2_ (PDB:1KQU). The purple ball is Ca^2+^, which coordinates with Asp48 and the glycine loop (Gly29, Gly31). The inhibitors are involved in π–π interactions with His6 and the catalytic His47.

As shown in Figure 9 for both GK181 and GK491, the naphthyl group is accommodated at the lipophilic pocket of the site and is involved in a T-shape interaction with His6. The carbonyl group points towards Ca^2+^ ion and is close enough to Gly29 to form a hydrogen bond. The extended heterocyclic aromatic system is involved in π–π interactions with the catalytic His47. These two models reproduce the key interactions, as known by co-crystallized ligands [31], and therefore may suggest the mode of interactions between either GK181 or GK491 and secreted sPLA_2_.

## 4. Conclusions

We present herein the synthesis of eight α-ketobenzothiazoles, using the reaction between benzothiazolyl lithium and the appropriate Weinreb amide as the key step. A series of α-ketobenzoxazoles, α-ketobenzimidazoles, and α-keto-1,2,4-oxadiazoles were also synthesized. All the synthetic heterocycles were evaluated for their ability to suppress the generation of PGE_2_ in renal mesangial cells after stimulation with IL-1 plus forskolin. Interestingly, two heterocycles were identified, which were found able to inhibit PGE_2_ formation at a nanomolar level. These structures may serve as leads for the development of novel potent inhibitors of PGE_2_ formation with potential anti-inflammatory and/or anticancer properties.

## Figures and Tables

**Table 1:** Compounds tested for their inhibition of PGE_2_ generation at 3 μM.

Entry.	Compound (Code Number)	Structure	% Inhibition (at 3 µM)
1	**8a** (GK181)	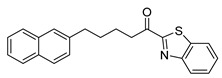	85
2	**8f** (GK517)	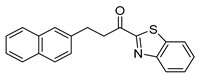	no inhibition
3	**8d** (GK489)	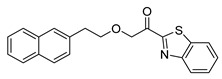	no inhibition
4	**12** (GK490)	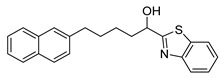	no inhibition
5	**17d** (GK491)	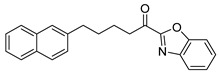	77
6	**17e** (GK492)	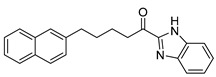	57
7	**8b** (GK299)	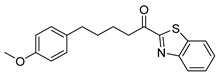	79
8	**17a** (GK355)	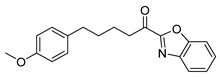	25
9	**17b** (GK358)	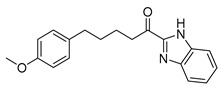	no inhibition
10	**22a** (GK367)	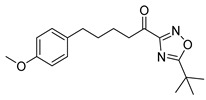	no inhibition
11	**22b** (GK368)	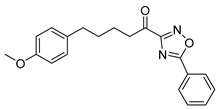	68
12	**22c** (GK369)	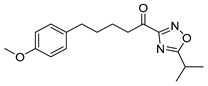	no inhibition
13	**17c** (GK453)	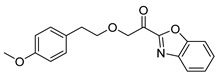	no inhibition
14	**8c** (GK455)	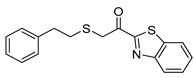	no inhibition
15	**8e** (GK516)	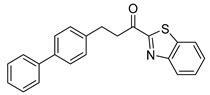	no inhibition
16	**8g** (GK518)	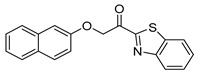	no inhibition
17	**8h** (GK519)	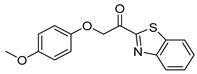	no inhibition

## Data Availability

The data presented in this study are available on request from the corresponding author. The data are not publicly available due to privacy.

## Supplementary Materials

The following are available online at https://www.mdpi.com/2218-273X/11/2/275/s1, Copies of ^1^H NMR and ^13^C NMR spectra of the final products.
