# Supplementary material for: α-Ketoheterocycles Able to Inhibit the Generation of Prostaglandin E2 (PGE2) in Rat Mesangial Cells
Source: Biomolecules. 2021 Feb 13;11(2):275. doi: 10.3390/biom11020275 (PMC7918003; doi:10.3390/biom11020275)
Supplement: Supplementary file 1 [file biomolecules-11-00275-s001.pdf]

α-Ketoheterocycles Able to Inhibit the Generation of Prostaglandin E_2 (PGE_2) in Rat Mesangial Cells

Anastasia Psarra [1,#], Maria A. Theodoropoulou [1,#], Martin Erhardt [2], Marina Mertiri [1], Christiana Mantzourani [1], Sofia Vasilakaki [1], Victoria Magrioti [1], Andrea Huwiler [2] and George Kokotos [1,*]

[1]*Laboratory of Organic Chemistry, Department of Chemistry, National and Kapodistrian University of Athens, Panepistimiopolis, Athens 15771, Greece*

[2]*Institute of Pharmacology, University of Bern, Bern 3010, Switzerland*

SUPPLEMENTARY MATERIALS

	Page
**NMR Spectra**	**2**

NMR Spectra

(8a)

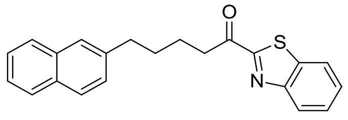

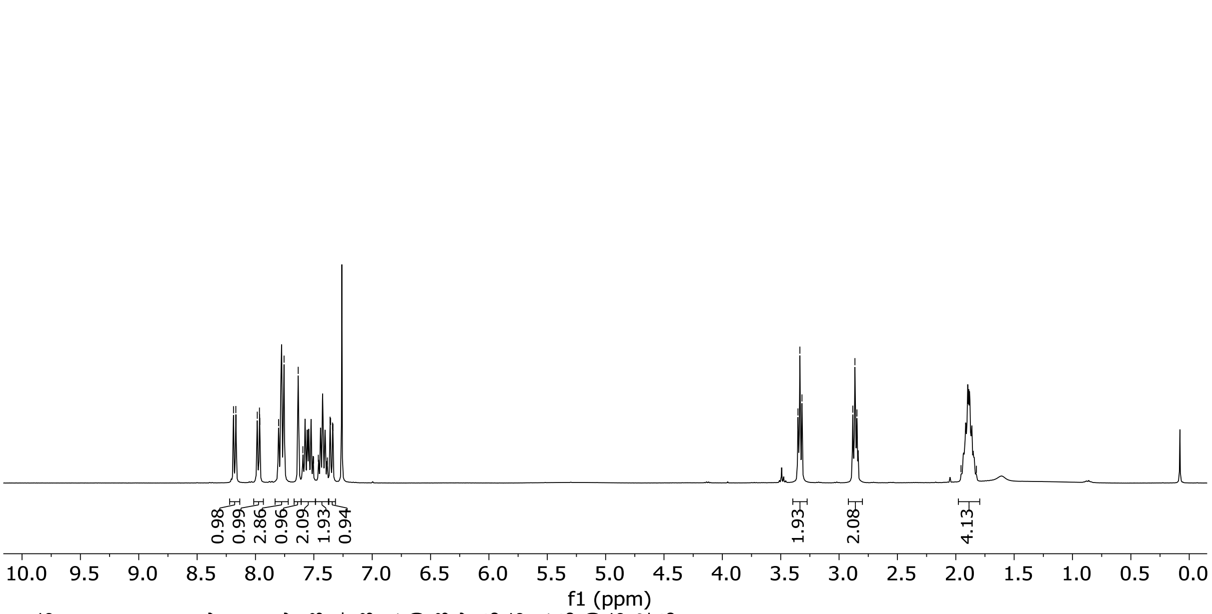

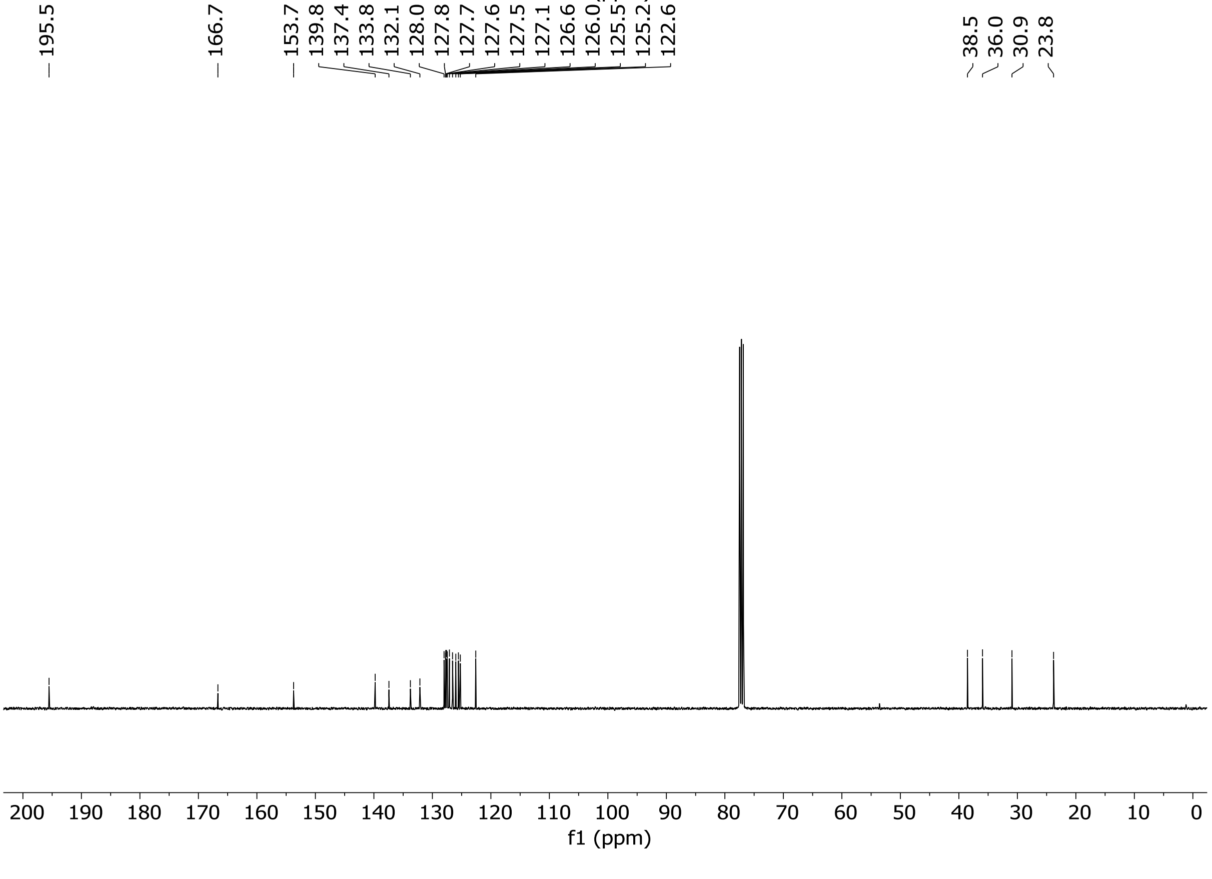

(8b)

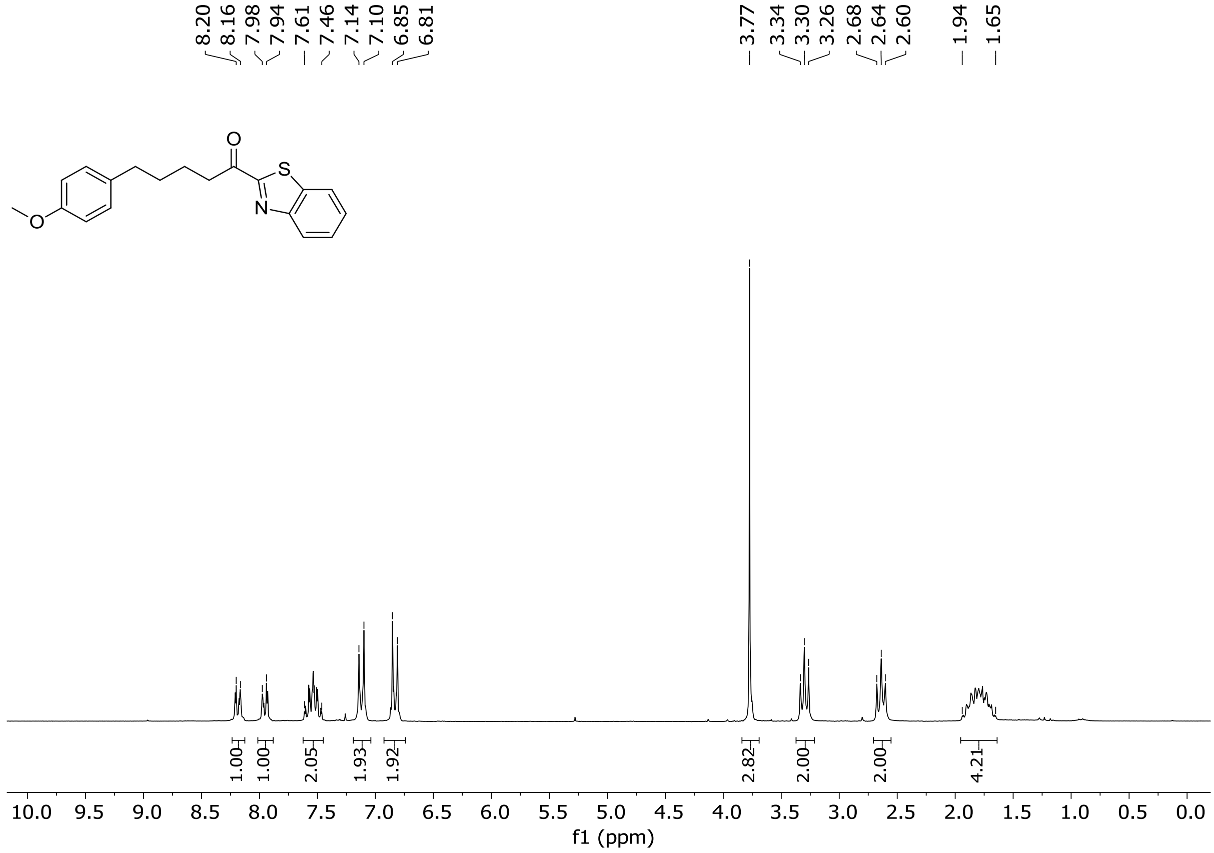
8.20
8.16
7.98
7.94
7.61
7.46
7.14
7.10
6.85
6.81
3.77
3.34
3.30
3.26
2.68
2.64
2.60
1.94
1.65
1.00
1.00
2.05
1.93
1.92
2.82
2.00
2.00
4.21
10.0 9.5 9.0 8.5 8.0 7.5 7.0 6.5 6.0 5.5 5.0 4.5 4.0 3.5 3.0 2.5 2.0 1.5 1.0 0.5 0.0
f1 (ppm)

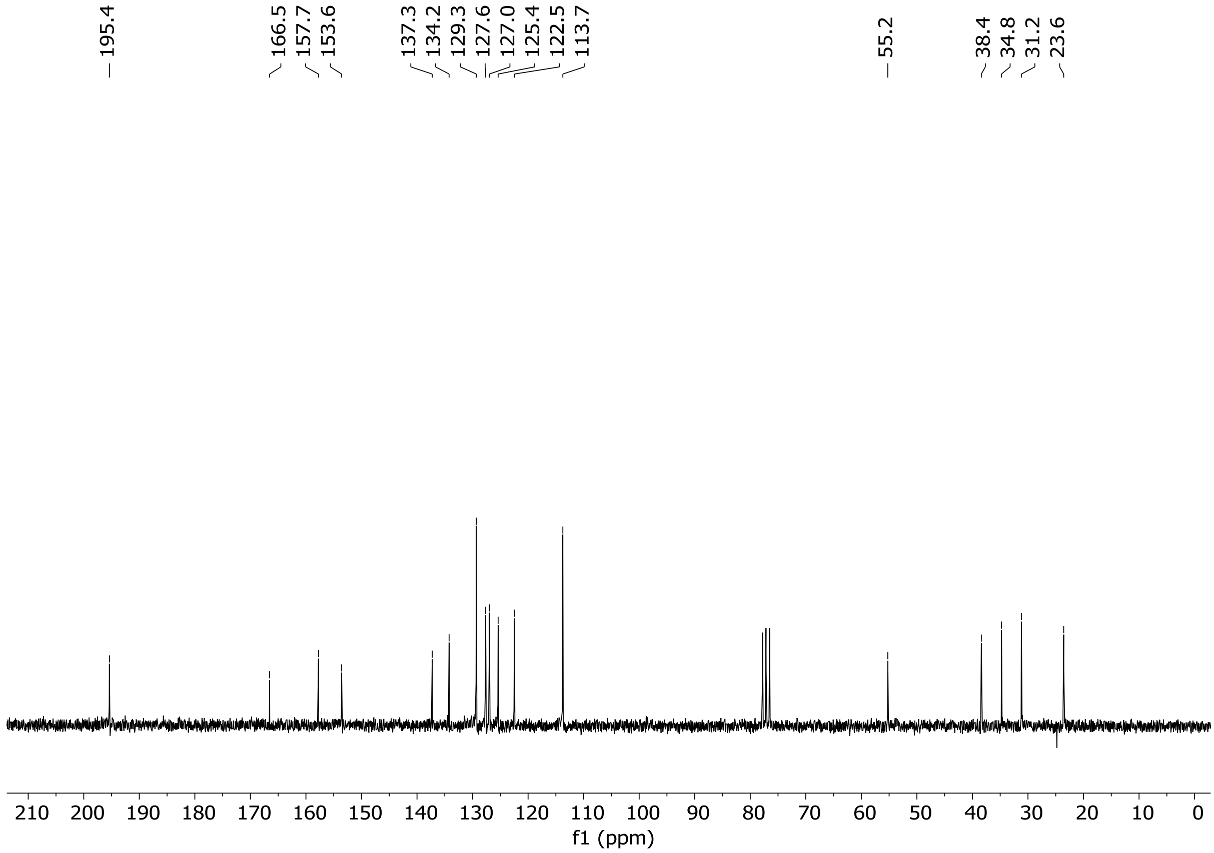
195.4
166.5
157.7
153.6
137.3
134.2
129.3
127.6
127.0
125.4
122.5
113.7
55.2
38.4
34.8
31.2
23.6
210 200 190 180 170 160 150 140 130 120 110 100 90 80 70 60 50 40 30 20 10 0
f1 (ppm)

(8c)

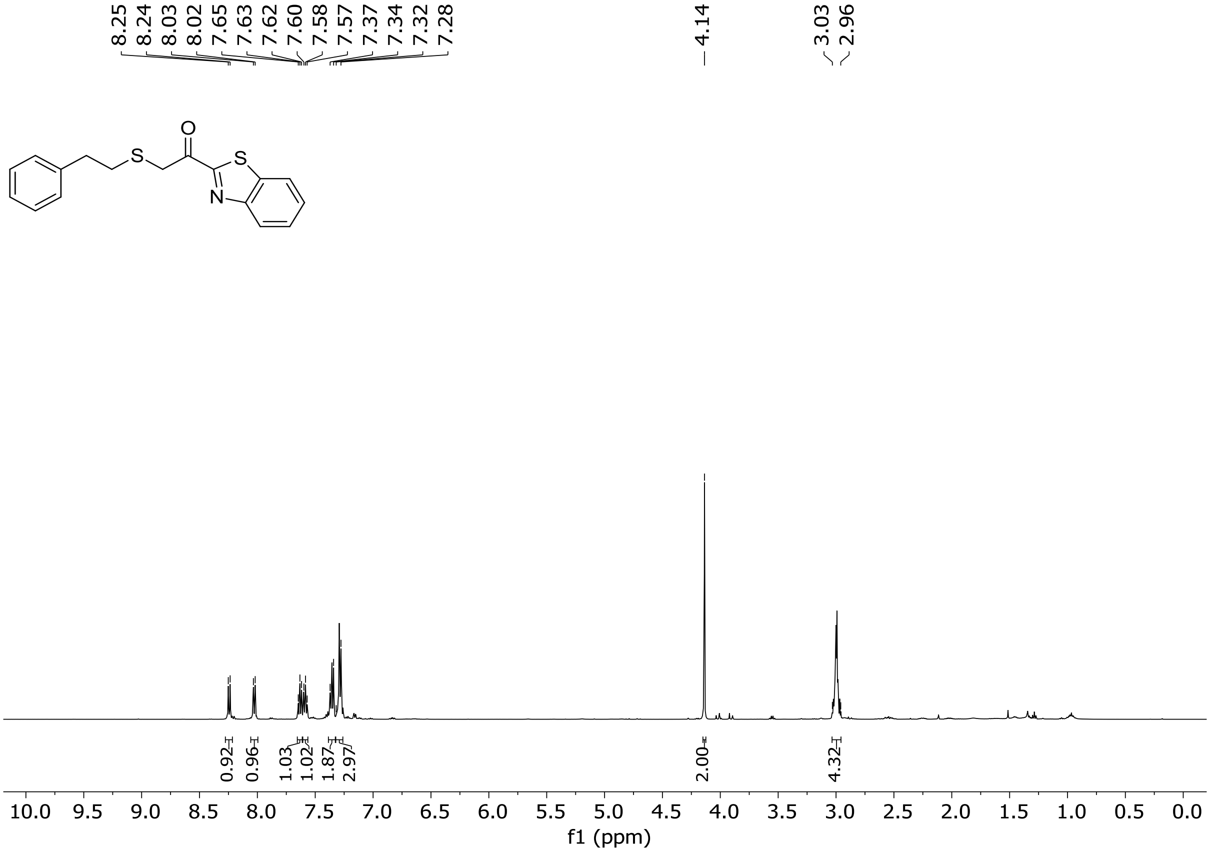
8.25
8.24
8.03
8.02
7.65
7.63
7.62
7.60
7.58
7.57
7.37
7.34
7.32
7.28
4.14
3.03
2.96
O
S
S
N
0.92
0.96
1.03
1.02
1.87
2.97
2.00
4.32
10.0 9.5 9.0 8.5 8.0 7.5 7.0 6.5 6.0 5.5 5.0 4.5 4.0 3.5 3.0 2.5 2.0 1.5 1.0 0.5 0.0
f1 (ppm)

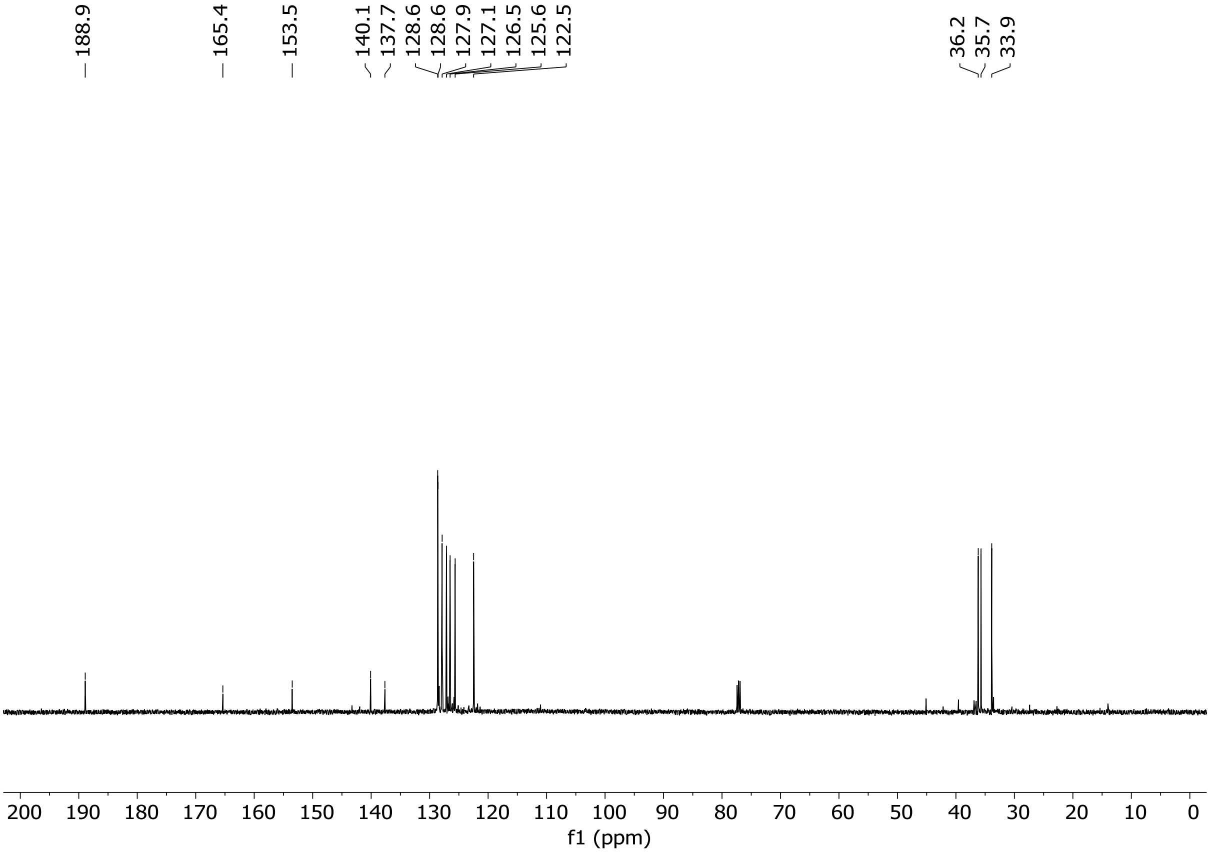
188.9
165.4
153.5
140.1
137.7
128.6
128.6
127.9
127.1
126.5
125.6
122.5
36.2
35.7
33.9
200 190 180 170 160 150 140 130 120 110 100 90 80 70 60 50 40 30 20 10 0
f1 (ppm)

(8d)

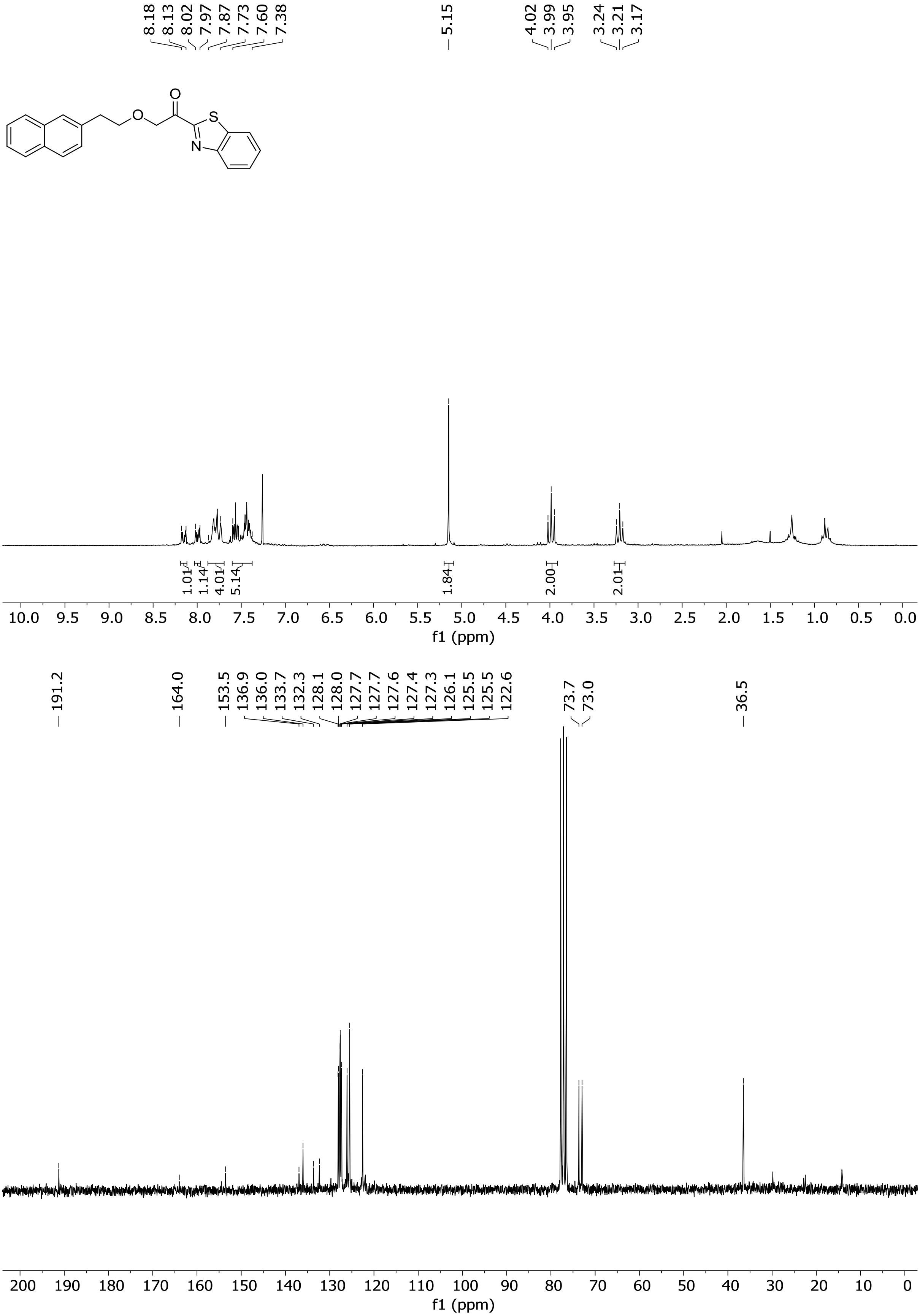
8.18 8.13 8.02 7.97 7.87 7.73 7.60 7.38
5.15
4.02 3.99 3.95
3.24 3.21 3.17
O
S
N
1.01 1.14 4.01 5.14
1.84
2.00
2.01
10.0 9.5 9.0 8.5 8.0 7.5 7.0 6.5 6.0 5.5 5.0 4.5 4.0 3.5 3.0 2.5 2.0 1.5 1.0 0.5 0.0
f1 (ppm)
191.2
164.0
153.5 136.9 136.0 133.7 132.3 128.1 128.0 127.7 127.7 127.6 127.4 127.3 126.1 125.5 125.5 122.6
73.7 73.0
36.5
200 190 180 170 160 150 140 130 120 110 100 90 80 70 60 50 40 30 20 10 0
f1 (ppm)

(8e)

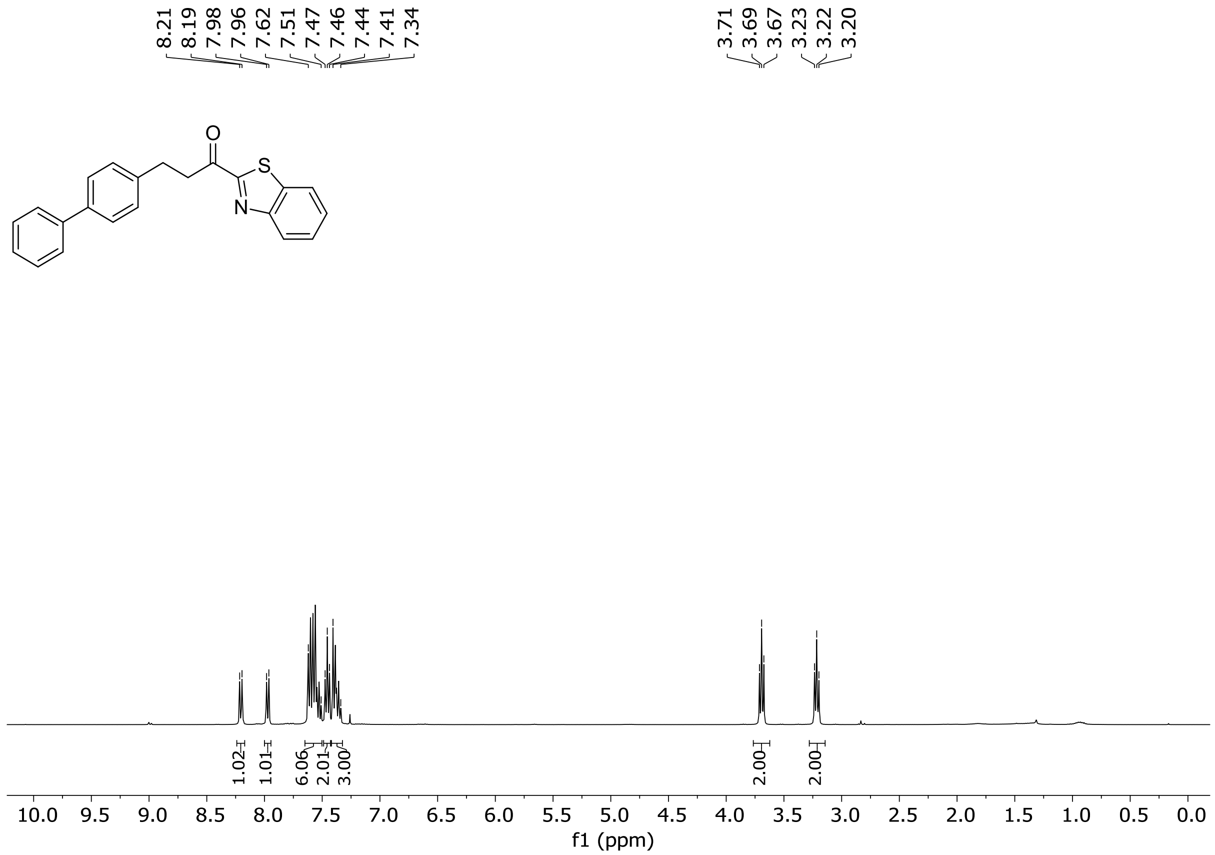
8.21
8.19
7.98
7.96
7.62
7.51
7.47
7.46
7.44
7.41
7.34
3.71
3.69
3.67
3.23
3.22
3.20
O
S
N
1.02
1.01
6.06
2.01
3.00
2.00
2.00
f1 (ppm)

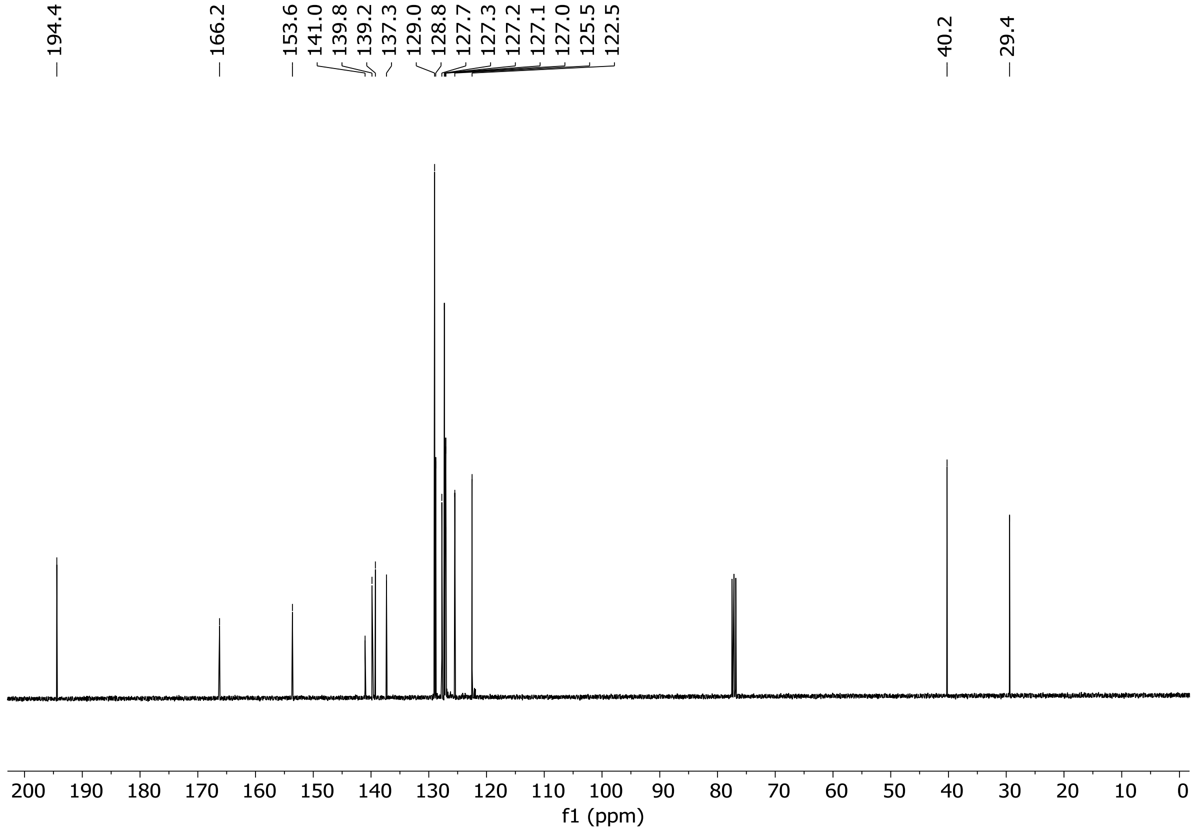
194.4
166.2
153.6
141.0
139.8
139.2
137.3
129.0
128.8
127.7
127.3
127.2
127.1
127.0
125.5
122.5
40.2
29.4
f1 (ppm)

(8f)

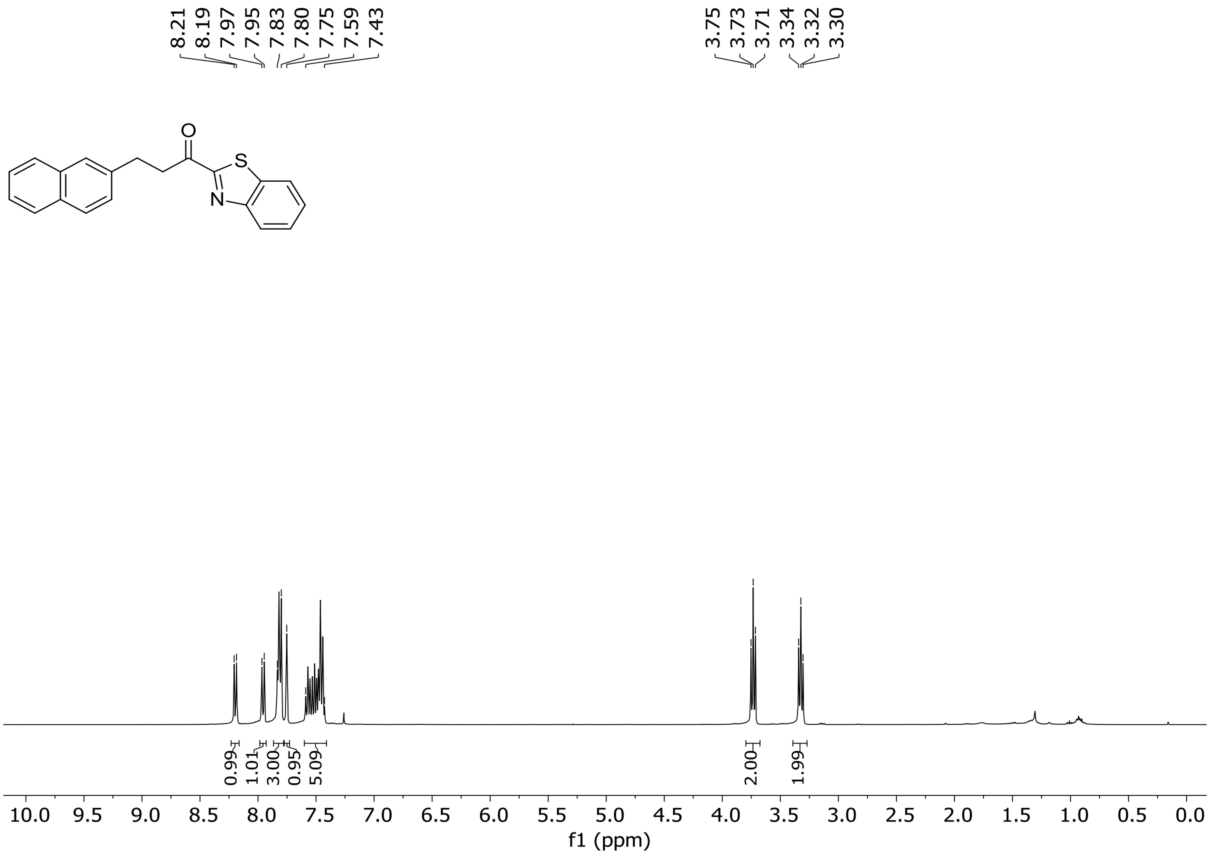
8.21
8.19
7.97
7.95
7.83
7.80
7.75
7.59
7.43
3.75
3.73
3.71
3.34
3.32
3.30
O
S
N
0.99
1.01
3.00
0.95
5.09
2.00
1.99
10.0 9.5 9.0 8.5 8.0 7.5 7.0 6.5 6.0 5.5 5.0 4.5 4.0 3.5 3.0 2.5 2.0 1.5 1.0 0.5 0.0
f1 (ppm)

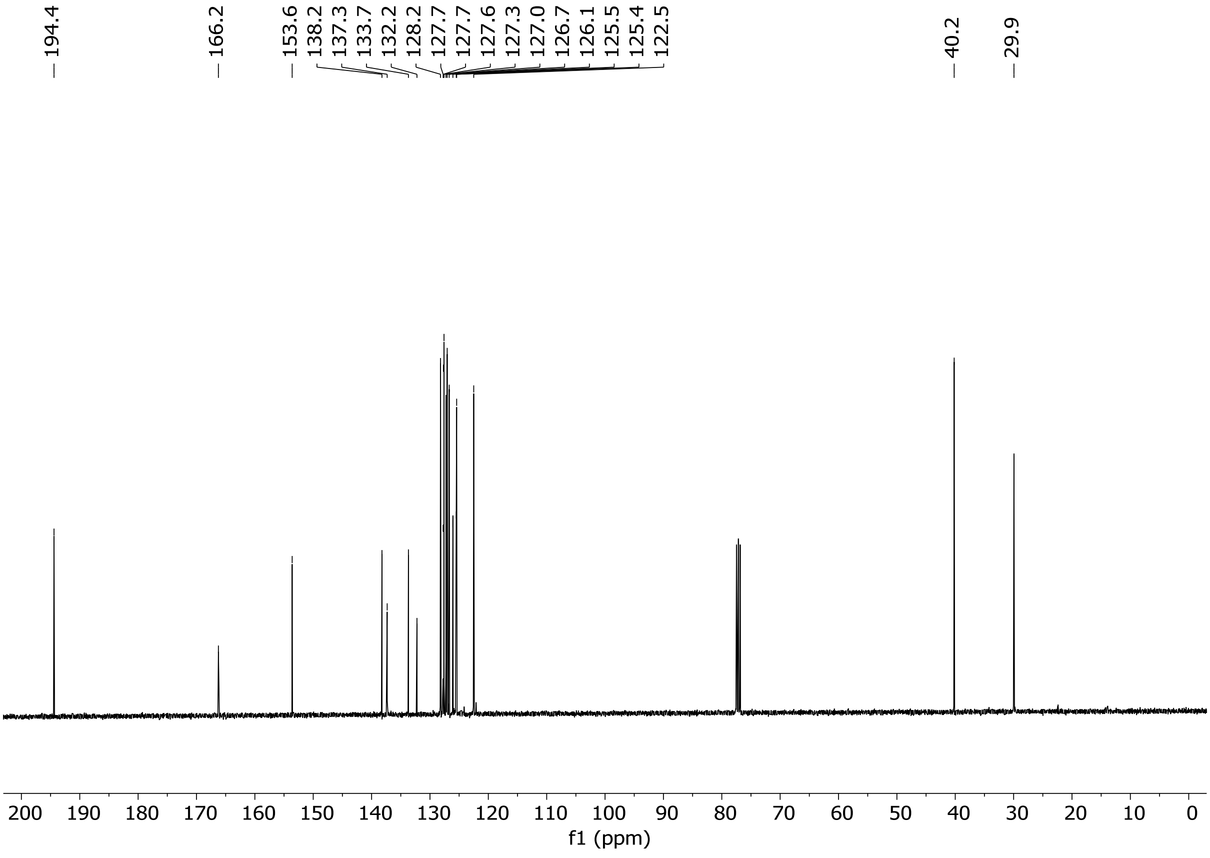
194.4
166.2
153.6
138.2
137.3
133.7
132.2
128.2
127.7
127.7
127.6
127.3
127.0
126.7
126.1
125.5
125.4
122.5
40.2
29.9
200 190 180 170 160 150 140 130 120 110 100 90 80 70 60 50 40 30 20 10 0
f1 (ppm)

(8g)

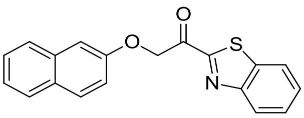
O
S
N

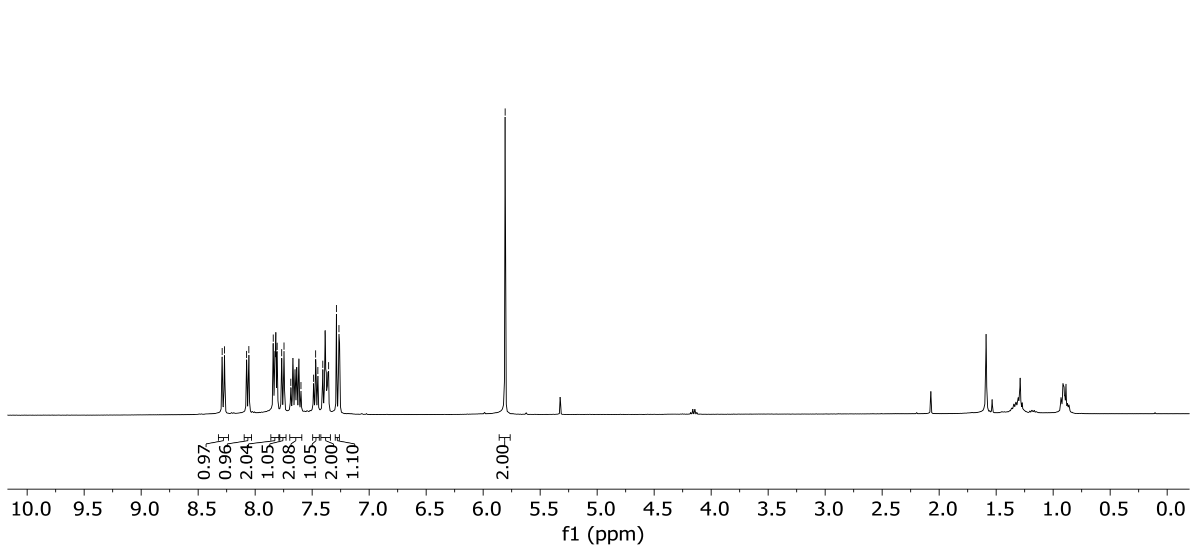
8.29
8.27
8.08
8.06
7.84
7.81
7.77
7.75
7.69
7.60
7.49
7.47
7.45
7.41
7.36
7.29
7.26
5.81
0.97
0.96
2.04
1.05
2.08
1.05
2.00
1.10
2.00
10.0 9.5 9.0 8.5 8.0 7.5 7.0 6.5 6.0 5.5 5.0 4.5 4.0 3.5 3.0 2.5 2.0 1.5 1.0 0.5 0.0
f1 (ppm)

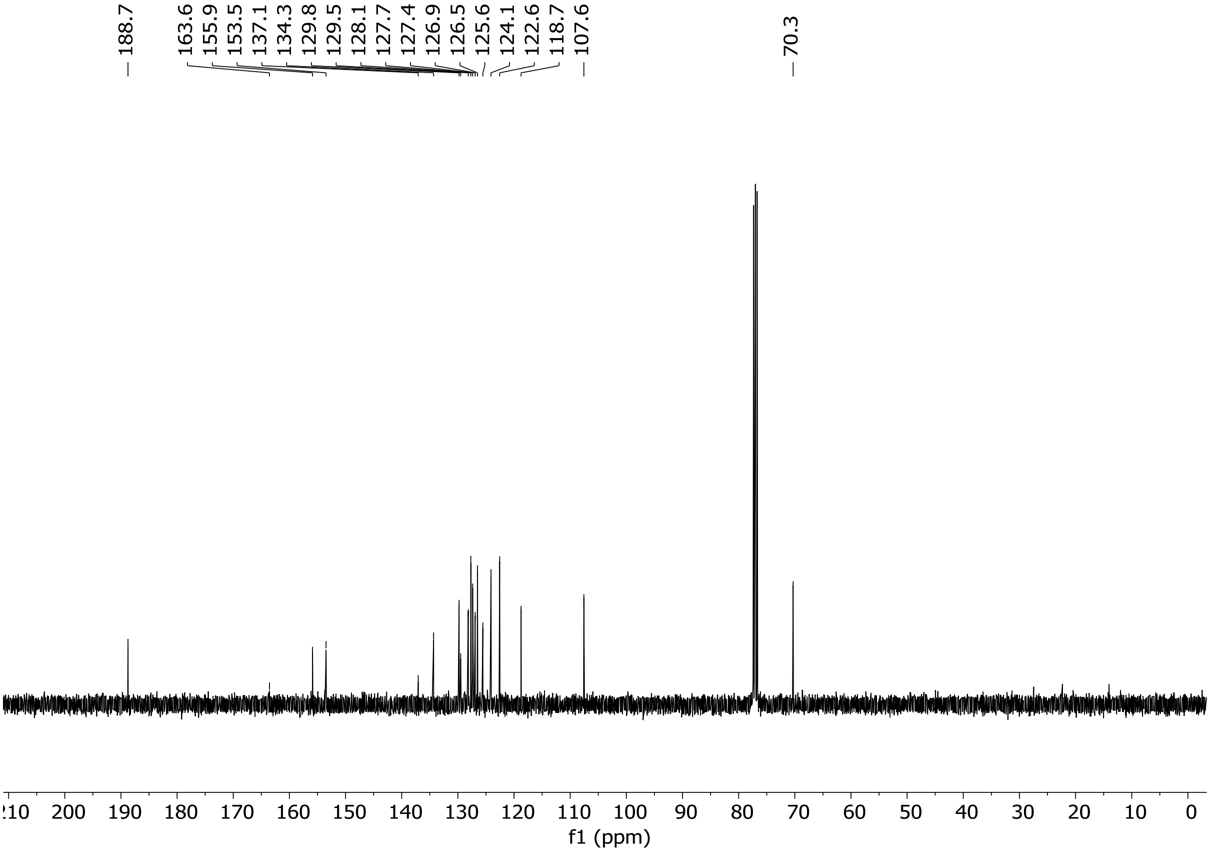
188.7
163.6
155.9
153.5
137.1
134.3
129.8
129.5
128.1
127.7
127.4
126.9
126.5
125.6
124.1
122.6
118.7
107.6
70.3
210 200 190 180 170 160 150 140 130 120 110 100 90 80 70 60 50 40 30 20 10 0
f1 (ppm)

(8h)

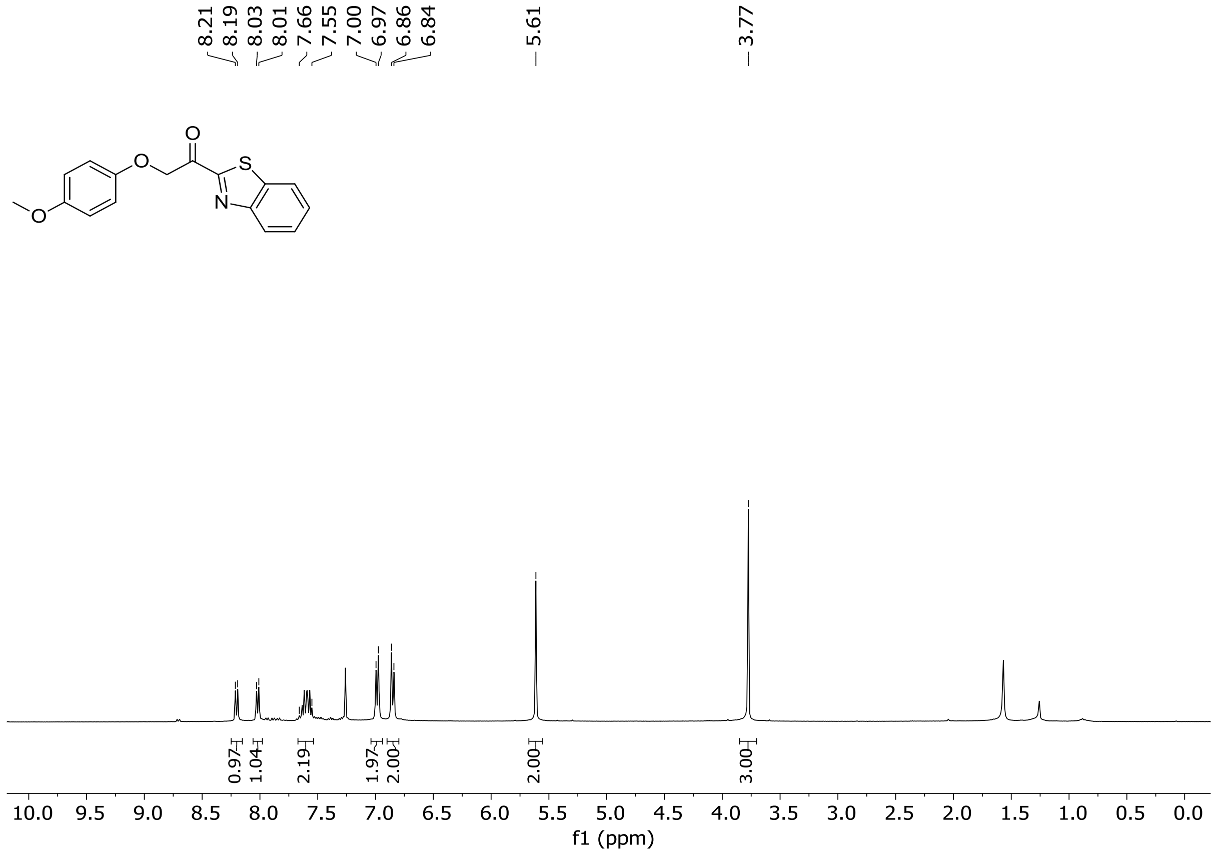
8.21
8.19
8.03
8.01
7.66
7.55
7.00
6.97
6.86
6.84
5.61
3.77
0.97
1.04
2.19
1.97
2.00
2.00
3.00
f1 (ppm)

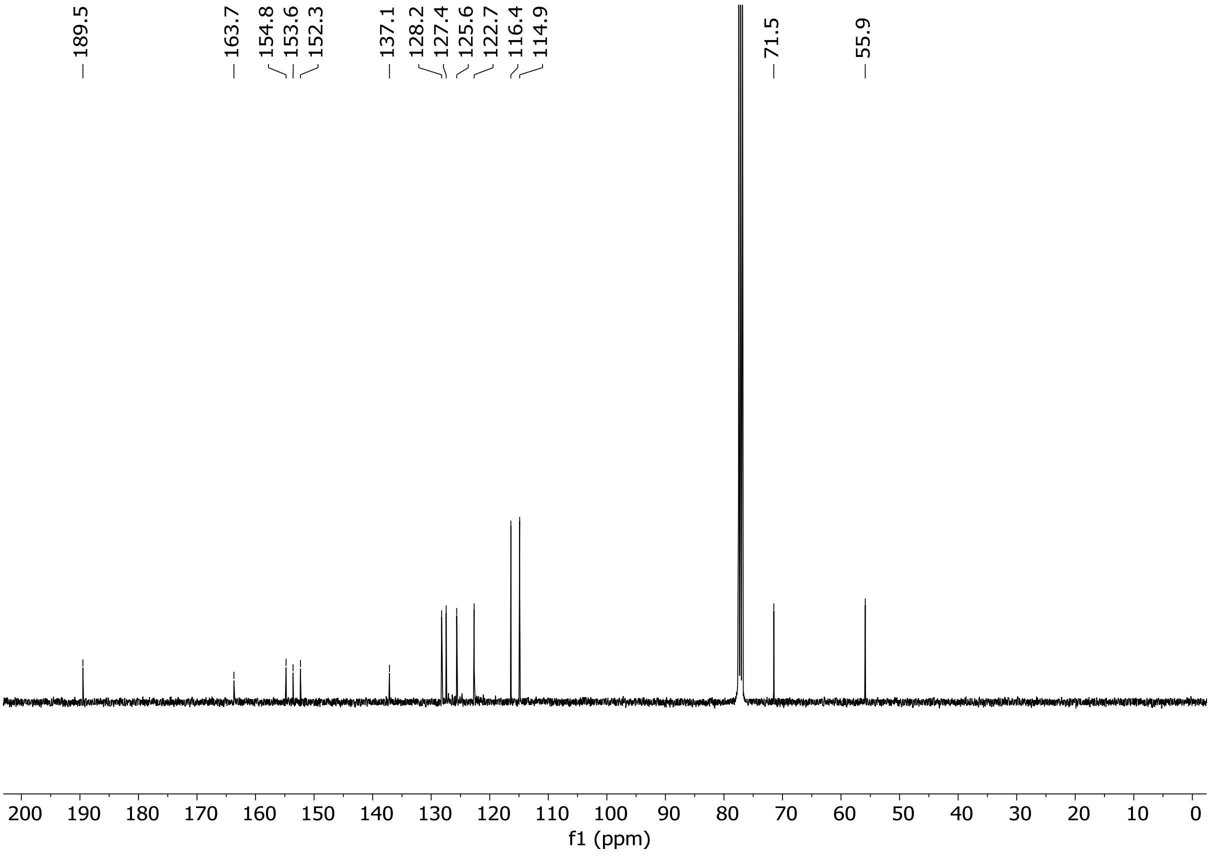
189.5
163.7
154.8
153.6
152.3
137.1
128.2
127.4
125.6
122.7
116.4
114.9
71.5
55.9
f1 (ppm)

(12)

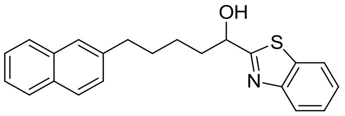
OH
S
N

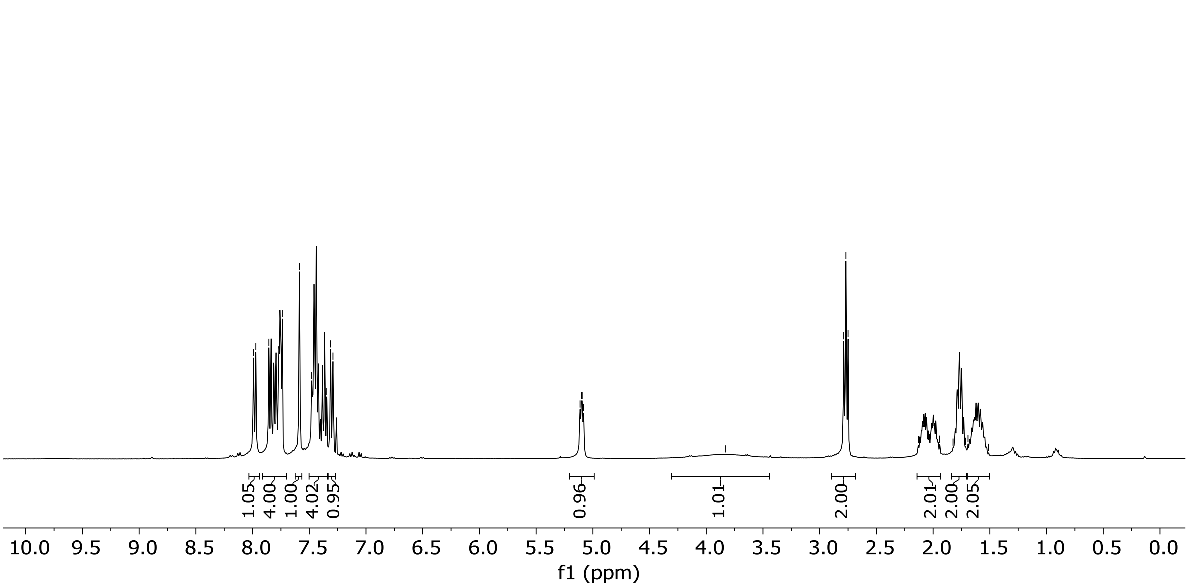
7.99 7.97 7.86 7.74 7.59 7.48 7.35 7.31 7.29
5.11 5.10 5.09 5.08
3.83
2.79 2.77 2.75 2.13 1.94 1.82 1.71 1.69 1.51
1.05 4.00 1.00 4.02 0.95
0.96
1.01
2.00
2.01 2.00 2.05
10.0 9.5 9.0 8.5 8.0 7.5 7.0 6.5 6.0 5.5 5.0 4.5 4.0 3.5 3.0 2.5 2.0 1.5 1.0 0.5 0.0
f1 (ppm)

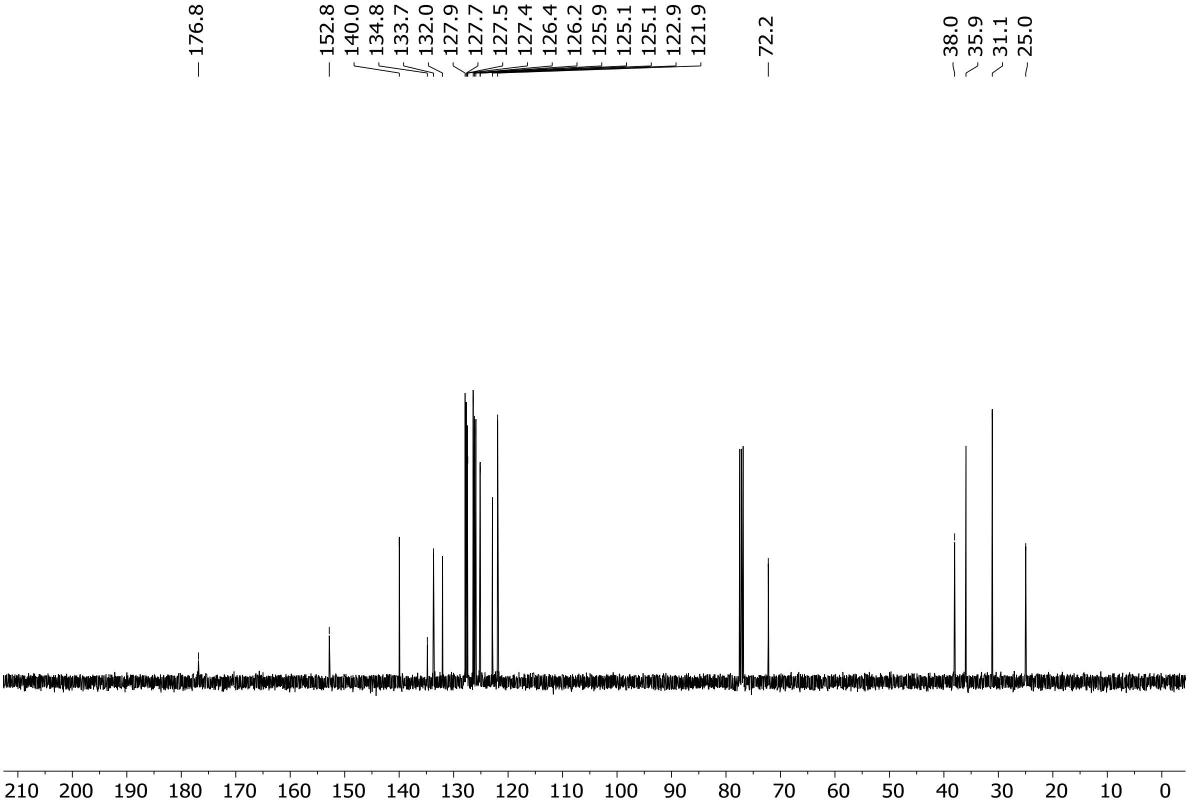
176.8
152.8 140.0 134.8 133.7 132.0 127.9 127.7 127.5 127.4 126.4 126.2 125.9 125.1 125.1 122.9 121.9
72.2
38.0 35.9 31.1 25.0
210 200 190 180 170 160 150 140 130 120 110 100 90 80 70 60 50 40 30 20 10 0
f1 (ppm)

(17a)

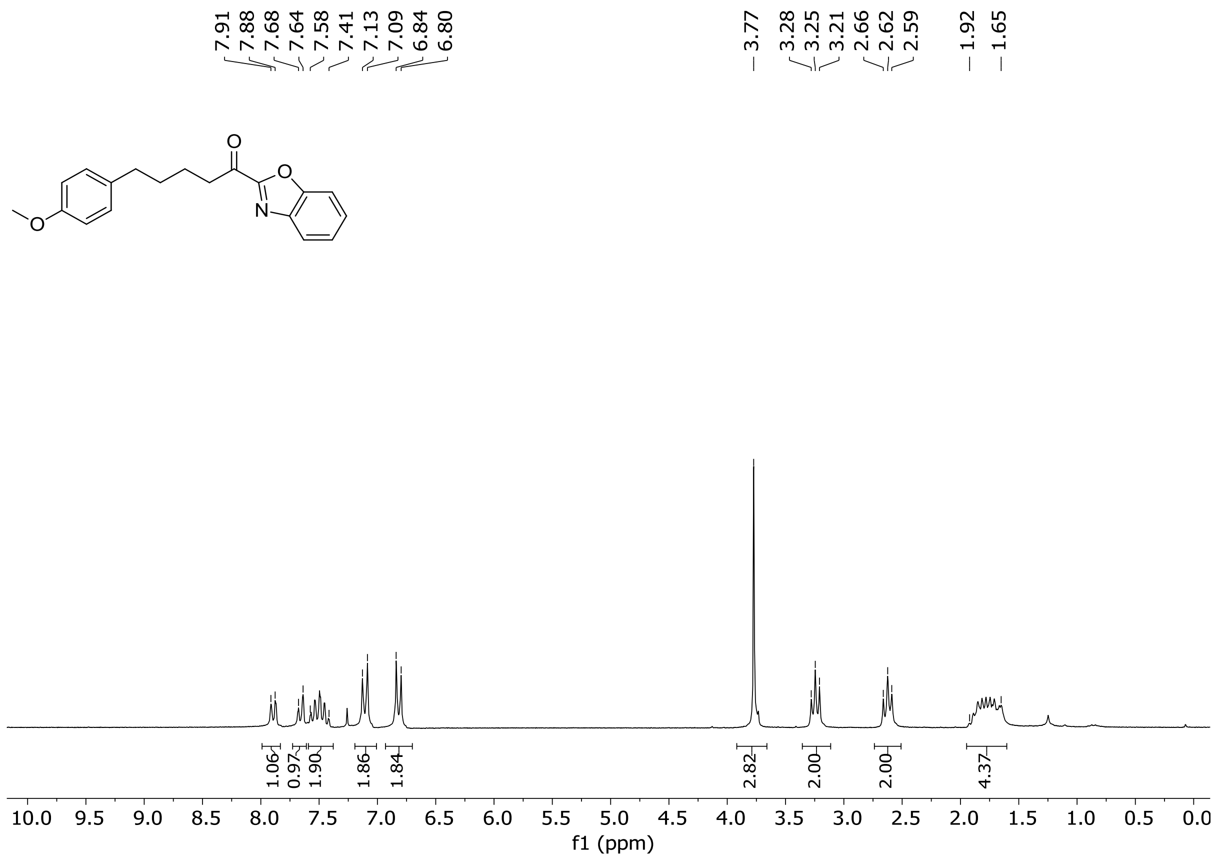
7.91 7.88 7.68 7.64 7.58 7.41 7.13 7.09 6.84 6.80
3.77 3.28 3.25 3.21 2.66 2.62 2.59 1.92 1.65
O
N
1.06 0.97 1.90 1.86 1.84 2.82 2.00 2.00 4.37
10.0 9.5 9.0 8.5 8.0 7.5 7.0 6.5 6.0 5.5 5.0 4.5 4.0 3.5 3.0 2.5 2.0 1.5 1.0 0.5 0.0
f1 (ppm)

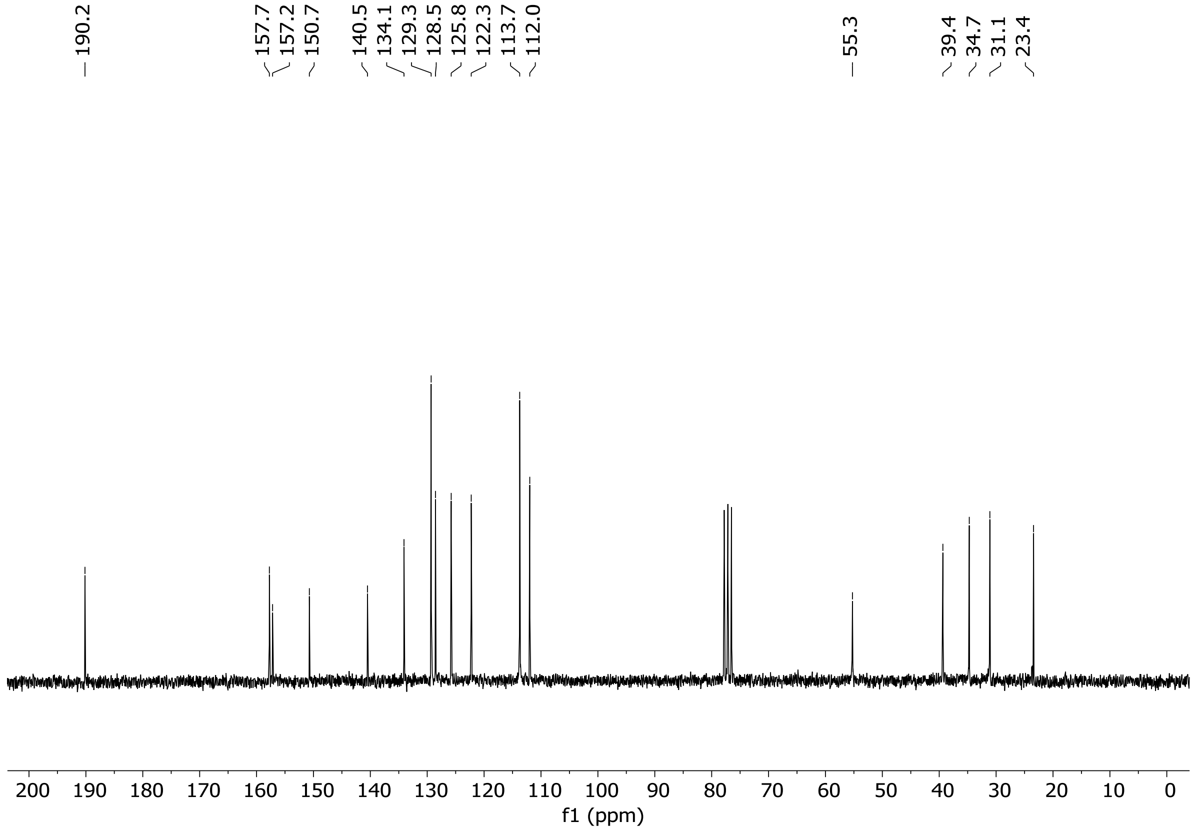
190.2
157.7 157.2 150.7 140.5 134.1 129.3 128.5 125.8 122.3 113.7 112.0
55.3
39.4 34.7 31.1 23.4
200 190 180 170 160 150 140 130 120 110 100 90 80 70 60 50 40 30 20 10 0
f1 (ppm)

(17b)

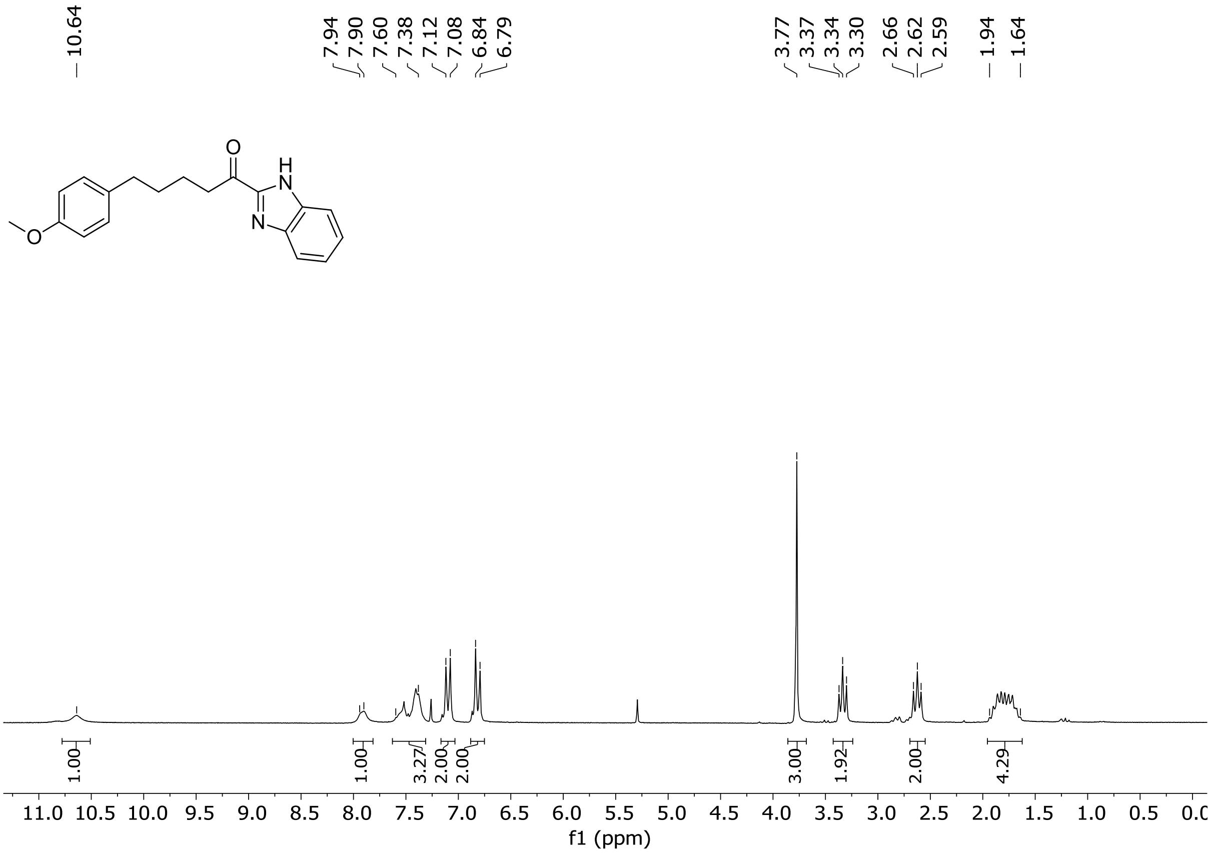
10.64
7.94
7.90
7.60
7.38
7.12
7.08
6.84
6.79
3.77
3.37
3.34
3.30
2.66
2.62
2.59
1.94
1.64
O
H
N
N
O
1.00
1.00
3.27
2.00
2.00
3.00
1.92
2.00
4.29
11.0 10.5 10.0 9.5 9.0 8.5 8.0 7.5 7.0 6.5 6.0 5.5 5.0 4.5 4.0 3.5 3.0 2.5 2.0 1.5 1.0 0.5 0.[illegible]
f1 (ppm)

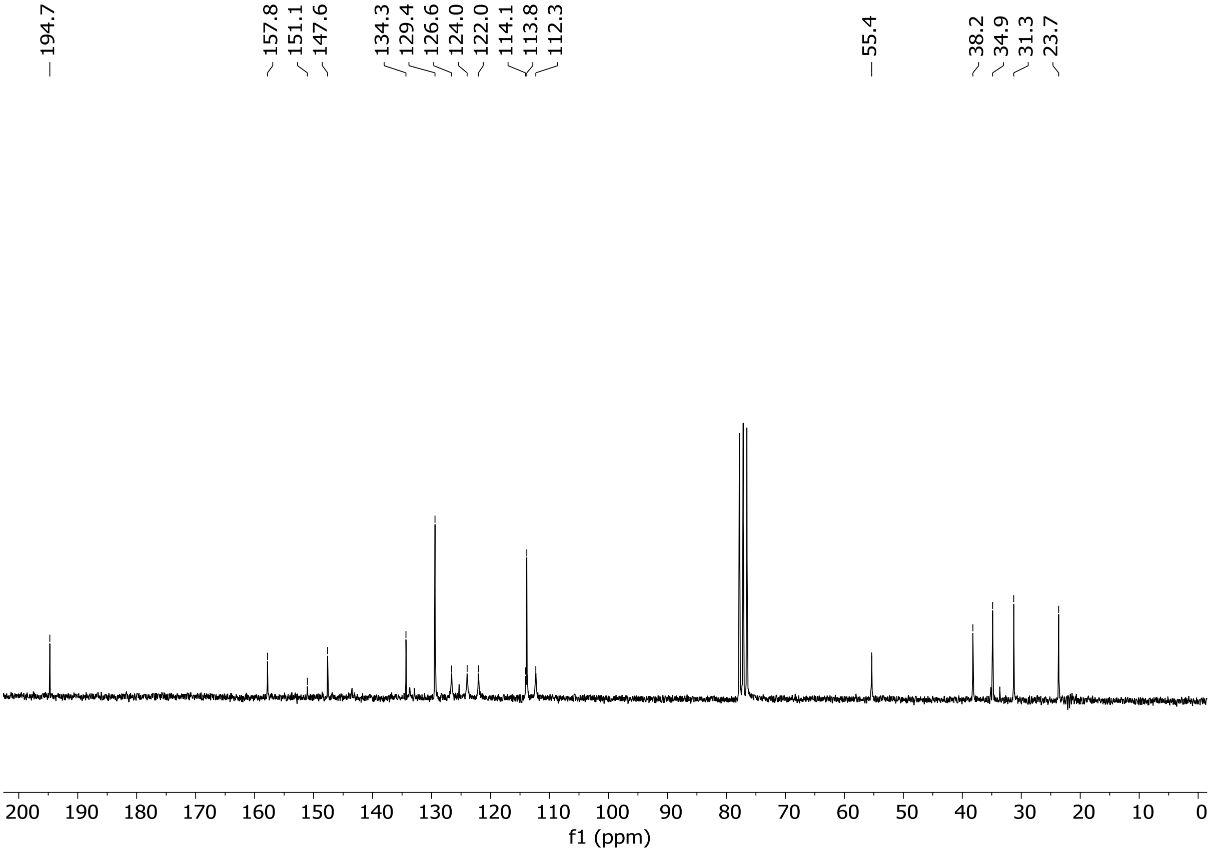
194.7
157.8
151.1
147.6
134.3
129.4
126.6
124.0
122.0
114.1
113.8
112.3
55.4
38.2
34.9
31.3
23.7
200 190 180 170 160 150 140 130 120 110 100 90 80 70 60 50 40 30 20 10 0
f1 (ppm)

(17c)

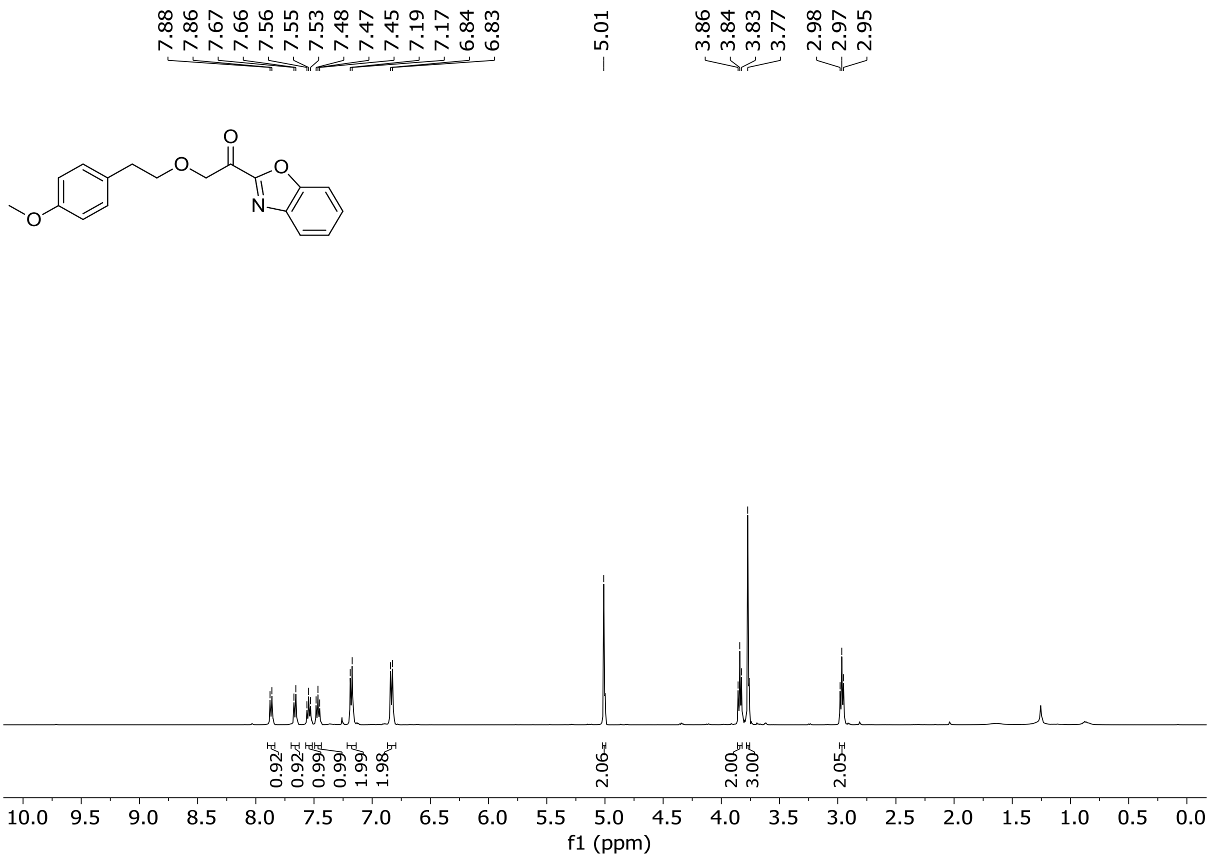
7.88 7.86 7.67 7.66 7.56 7.55 7.53 7.48 7.47 7.45 7.19 7.17 6.84 6.83
5.01
3.86 3.84 3.83 3.77
2.98 2.97 2.95
O
O
O
N
O
0.92 0.92 0.99 0.99 1.99 1.98
2.06
2.00 3.00
2.05
10.0 9.5 9.0 8.5 8.0 7.5 7.0 6.5 6.0 5.5 5.0 4.5 4.0 3.5 3.0 2.5 2.0 1.5 1.0 0.5 0.0
f1 (ppm)

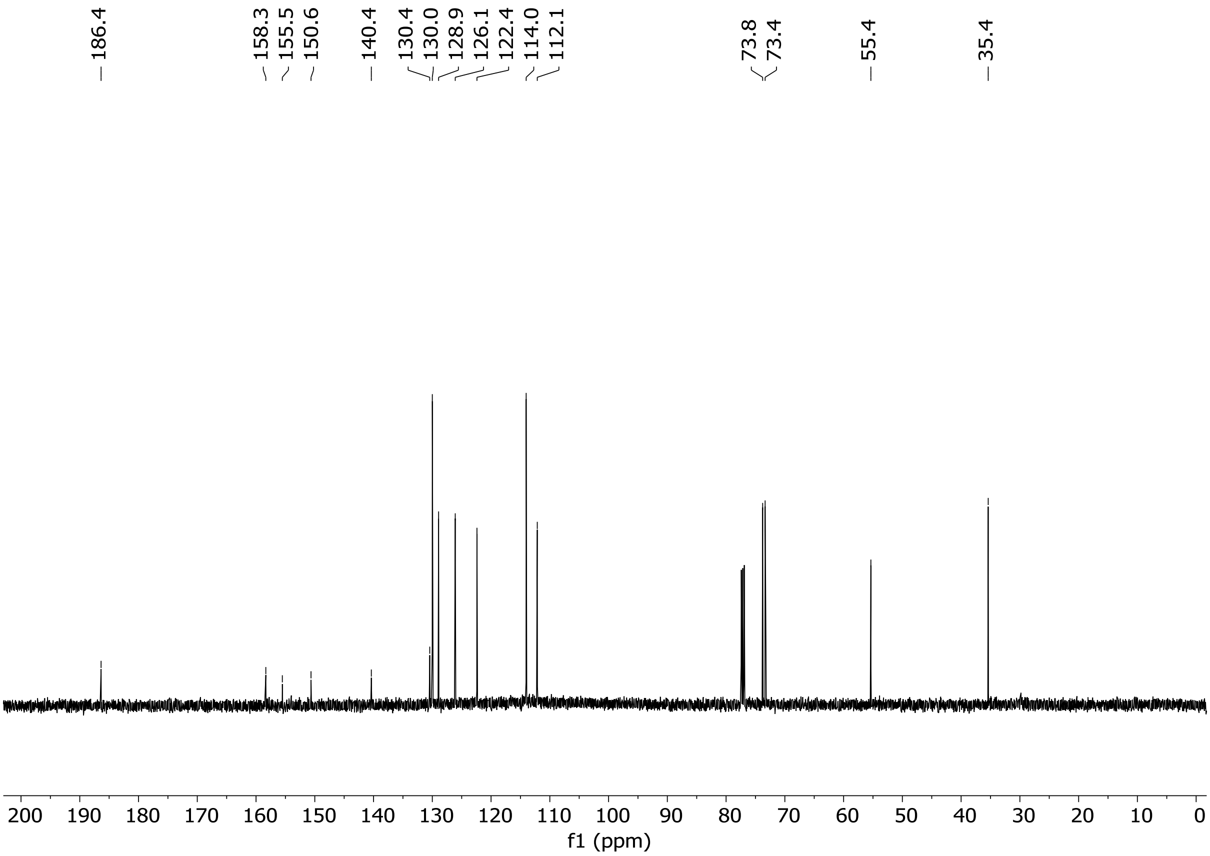
186.4
158.3 155.5 150.6
140.4
130.4 130.0 128.9 126.1 122.4 114.0 112.1
73.8 73.4
55.4
35.4
200 190 180 170 160 150 140 130 120 110 100 90 80 70 60 50 40 30 20 10 0
f1 (ppm)

(17d)

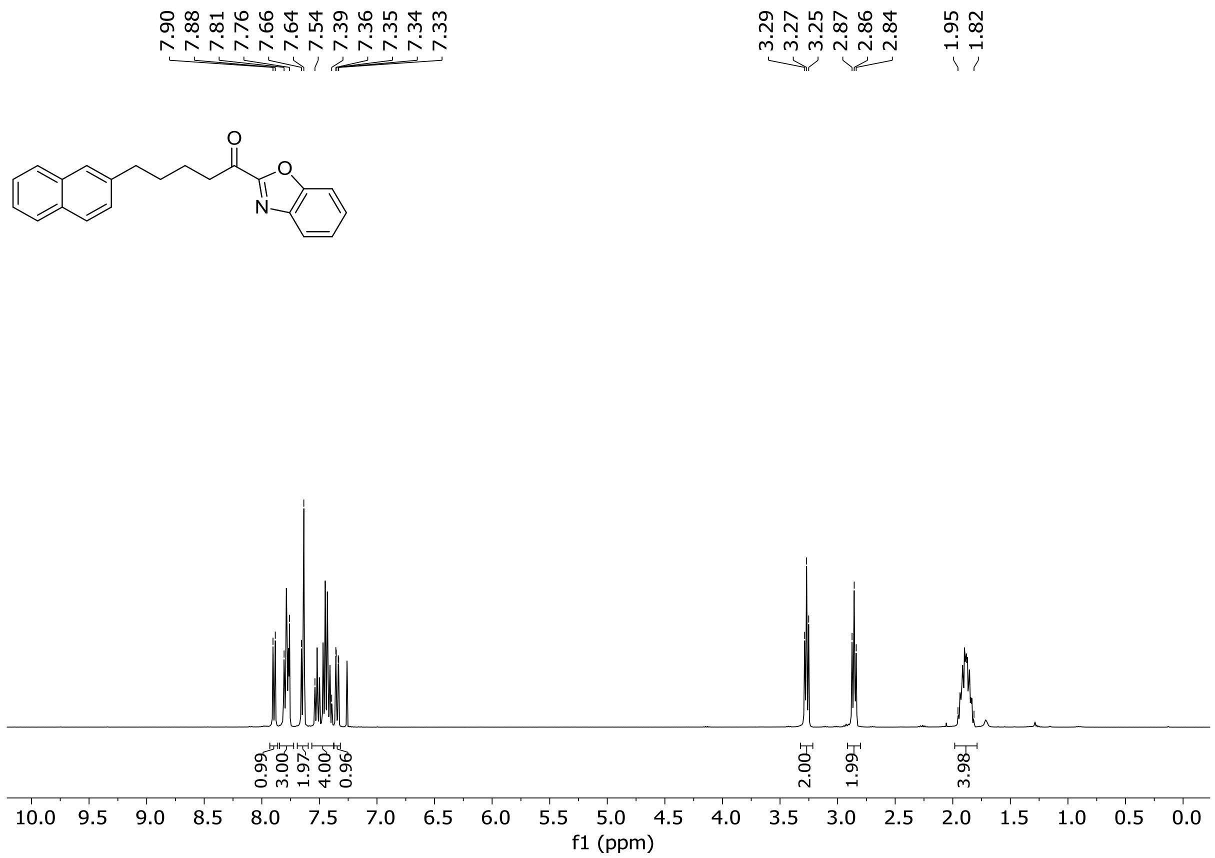
7.90
7.88
7.81
7.76
7.66
7.64
7.54
7.39
7.36
7.35
7.34
7.33
3.29
3.27
3.25
2.87
2.86
2.84
1.95
1.82
O
N
0.99
3.00
1.97
4.00
0.96
2.00
1.99
3.98
10.0 9.5 9.0 8.5 8.0 7.5 7.0 6.5 6.0 5.5 5.0 4.5 4.0 3.5 3.0 2.5 2.0 1.5 1.0 0.5 0.0
f1 (ppm)

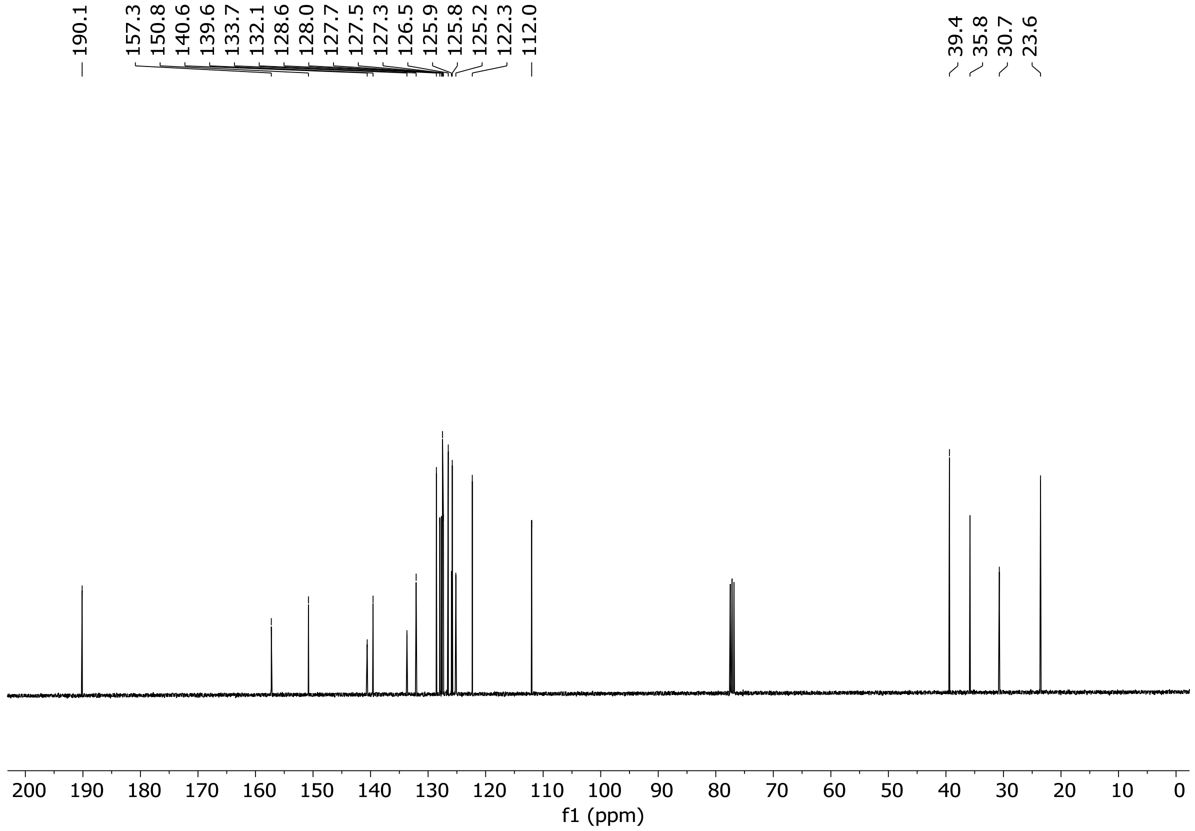
190.1
157.3
150.8
140.6
139.6
133.7
132.1
128.6
128.0
127.7
127.5
127.3
126.5
125.9
125.8
125.2
122.3
112.0
39.4
35.8
30.7
23.6
200 190 180 170 160 150 140 130 120 110 100 90 80 70 60 50 40 30 20 10 0
f1 (ppm)

(17e)

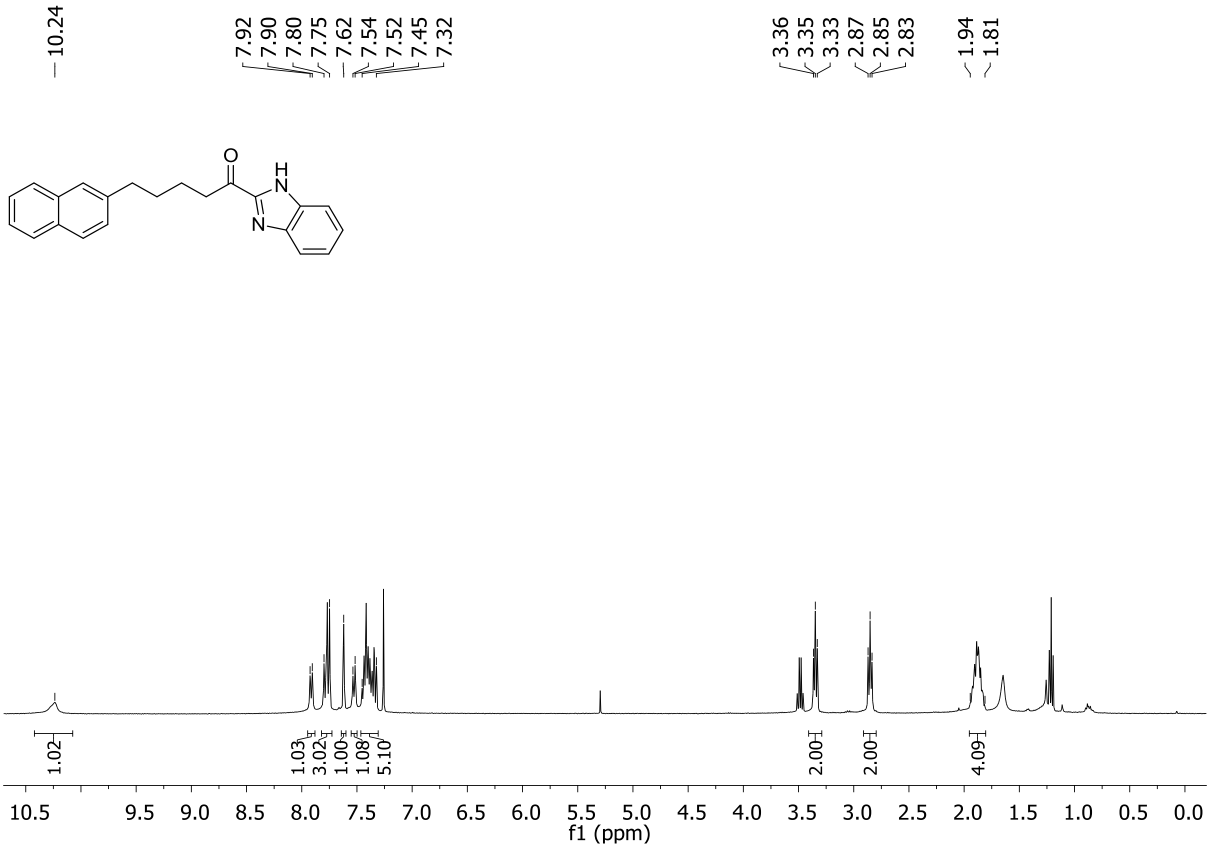
10.24
7.92
7.90
7.80
7.75
7.62
7.54
7.52
7.45
7.32
3.36
3.35
3.33
2.87
2.85
2.83
1.94
1.81
O
H
N
N
1.02
1.03
3.02
1.00
1.08
5.10
2.00
2.00
4.09
10.5 9.5 9.0 8.5 8.0 7.5 7.0 6.5 6.0 5.5 5.0 4.5 4.0 3.5 3.0 2.5 2.0 1.5 1.0 0.5 0.0
f1 (ppm)

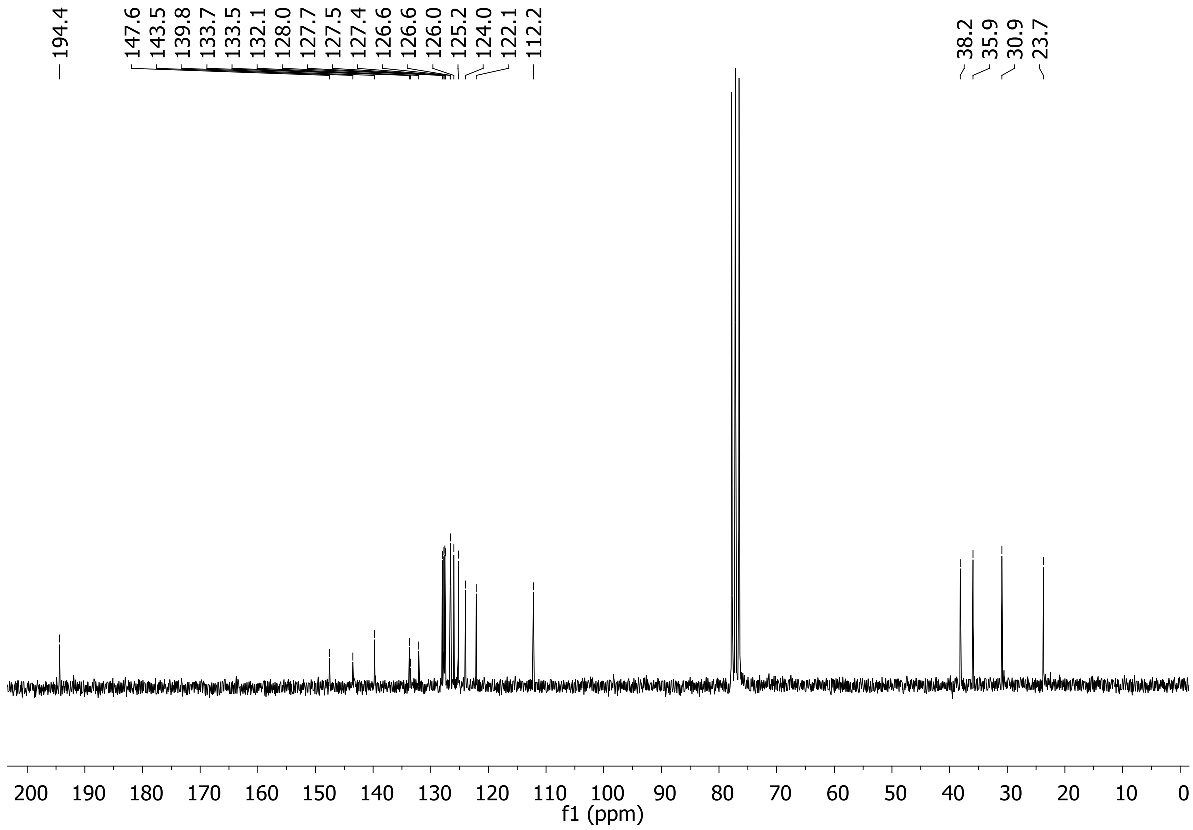
194.4
147.6
143.5
139.8
133.7
133.5
132.1
128.0
127.7
127.5
127.4
126.6
126.6
126.0
125.2
124.0
122.1
112.2
38.2
35.9
30.9
23.7
200 190 180 170 160 150 140 130 120 110 100 90 80 70 60 50 40 30 20 10 0
f1 (ppm)

(22a)

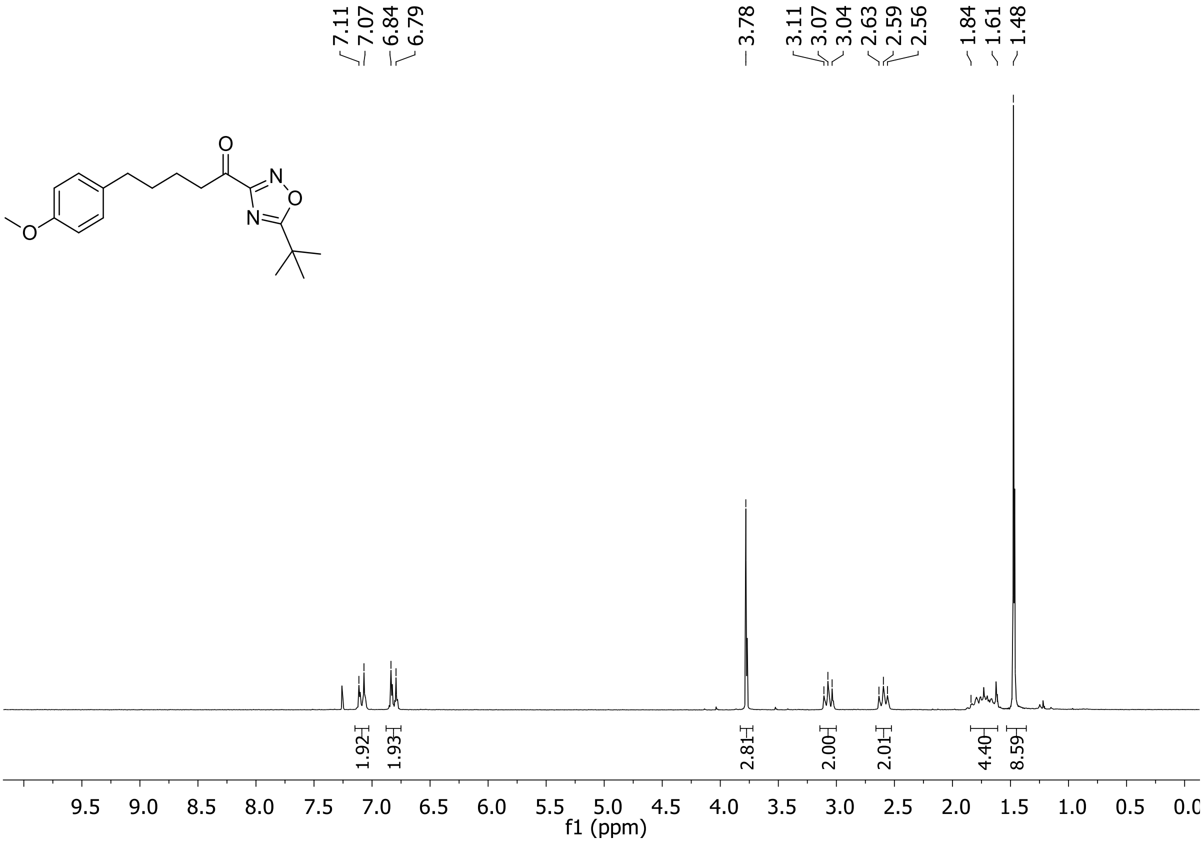
O
N
O
N
O
7.11
7.07
6.84
6.79
3.78
3.11
3.07
3.04
2.63
2.59
2.56
1.84
1.61
1.48
1.92
1.93
2.81
2.00
2.01
4.40
8.59
9.5 9.0 8.5 8.0 7.5 7.0 6.5 6.0 5.5 5.0 4.5 4.0 3.5 3.0 2.5 2.0 1.5 1.0 0.5 0.0
f1 (ppm)

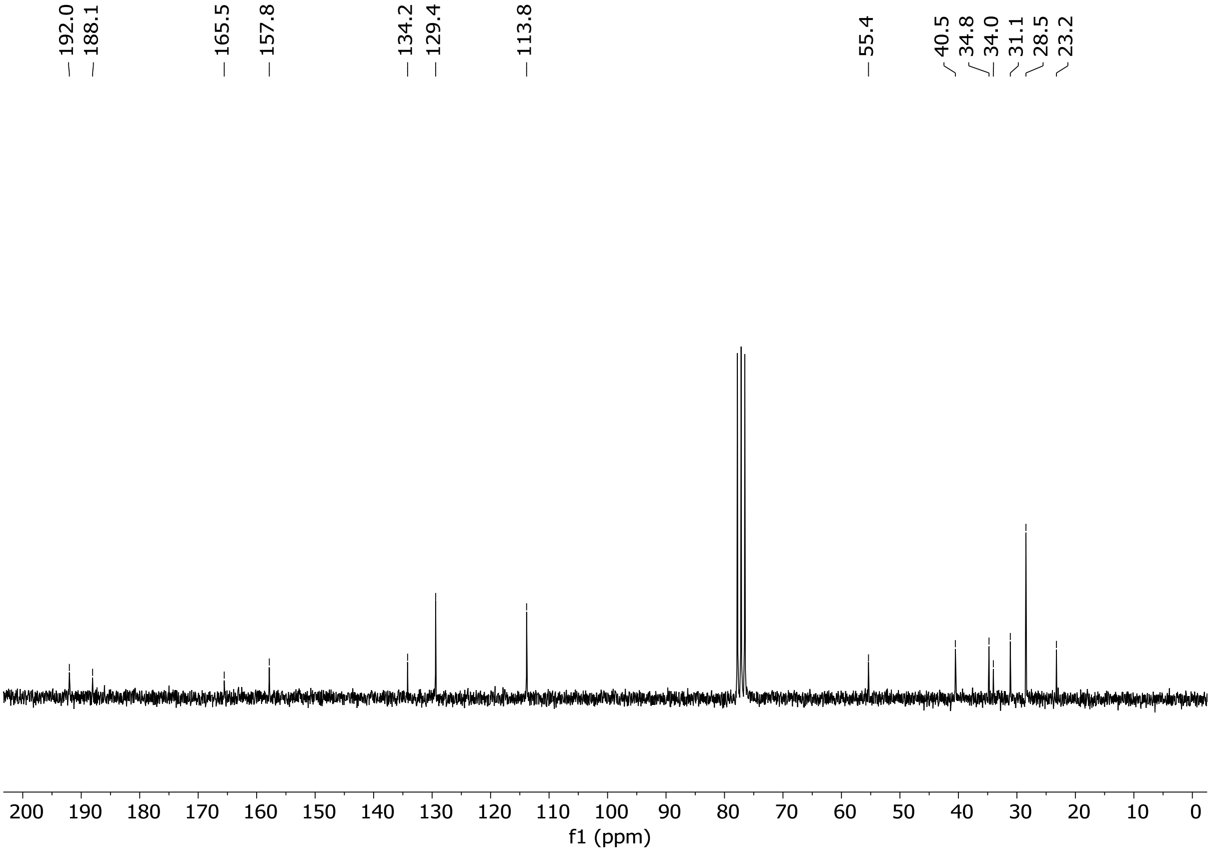
192.0
188.1
165.5
157.8
134.2
129.4
113.8
55.4
40.5
34.8
34.0
31.1
28.5
23.2
200 190 180 170 160 150 140 130 120 110 100 90 80 70 60 50 40 30 20 10 0
f1 (ppm)

(22b)

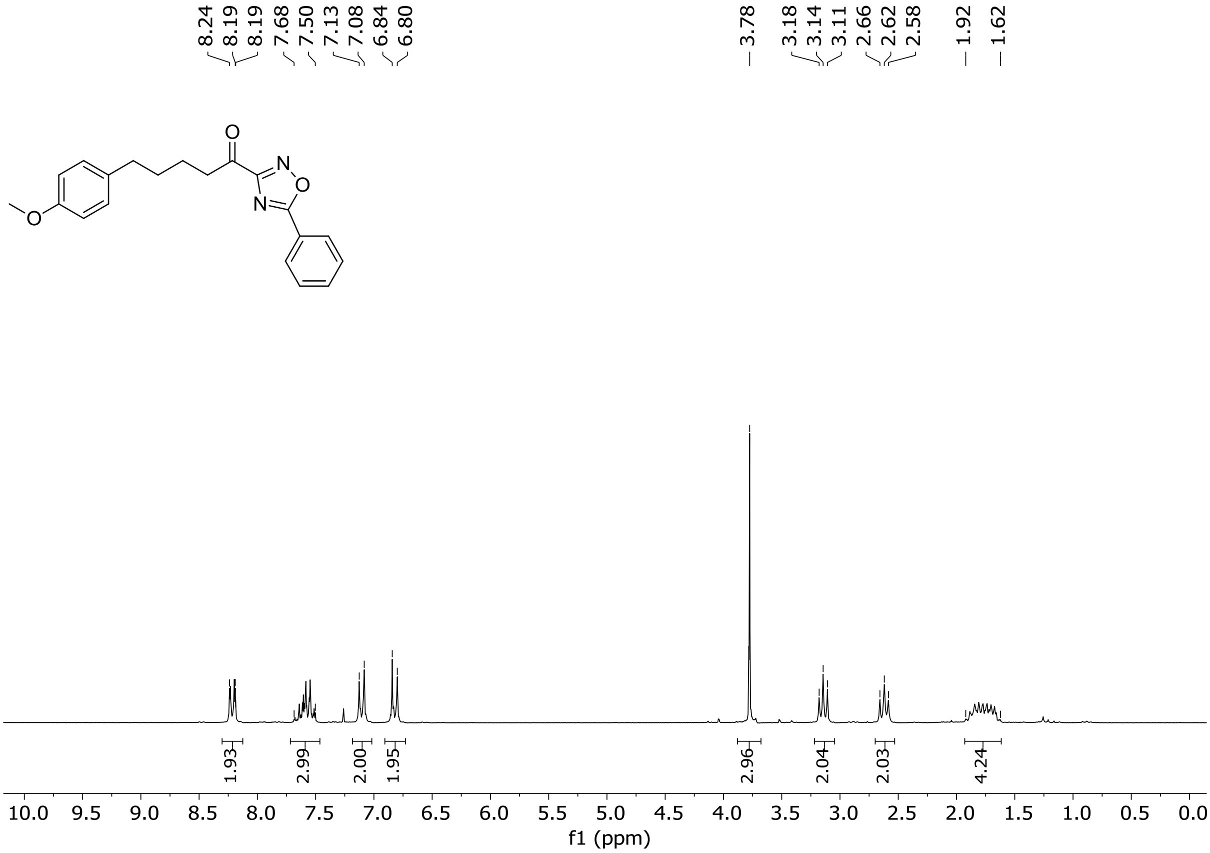
8.24 8.19 8.19 7.68 7.50 7.13 7.08 6.84 6.80
3.78
3.18 3.14 3.11 2.66 2.62 2.58
1.92 1.62
O
N
O
N
O
1.93 2.99 2.00 1.95 2.96 2.04 2.03 4.24
10.0 9.5 9.0 8.5 8.0 7.5 7.0 6.5 6.0 5.5 5.0 4.5 4.0 3.5 3.0 2.5 2.0 1.5 1.0 0.5 0.0
f1 (ppm)

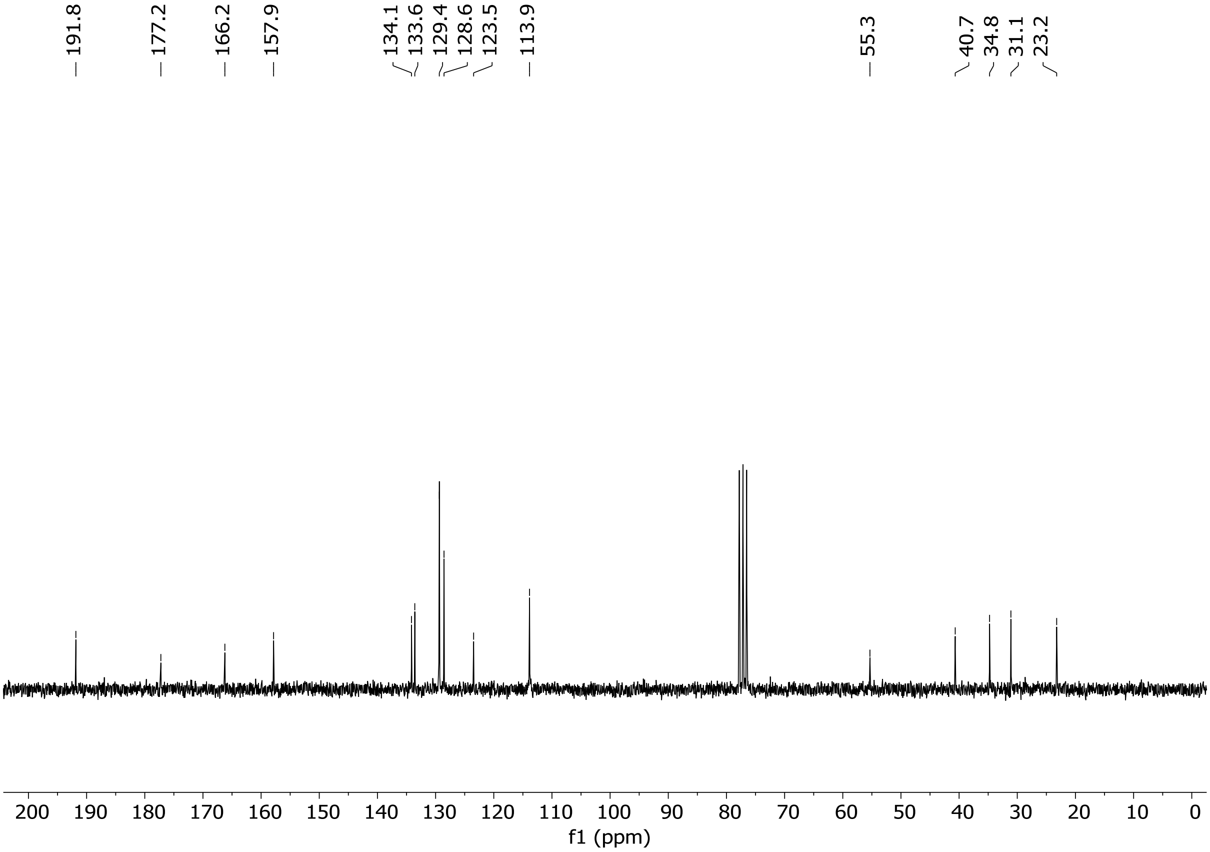
191.8 177.2 166.2 157.9
134.1 133.6 129.4 128.6 123.5 113.9
55.3
40.7 34.8 31.1 23.2
200 190 180 170 160 150 140 130 120 110 100 90 80 70 60 50 40 30 20 10 0
f1 (ppm)

(22c)

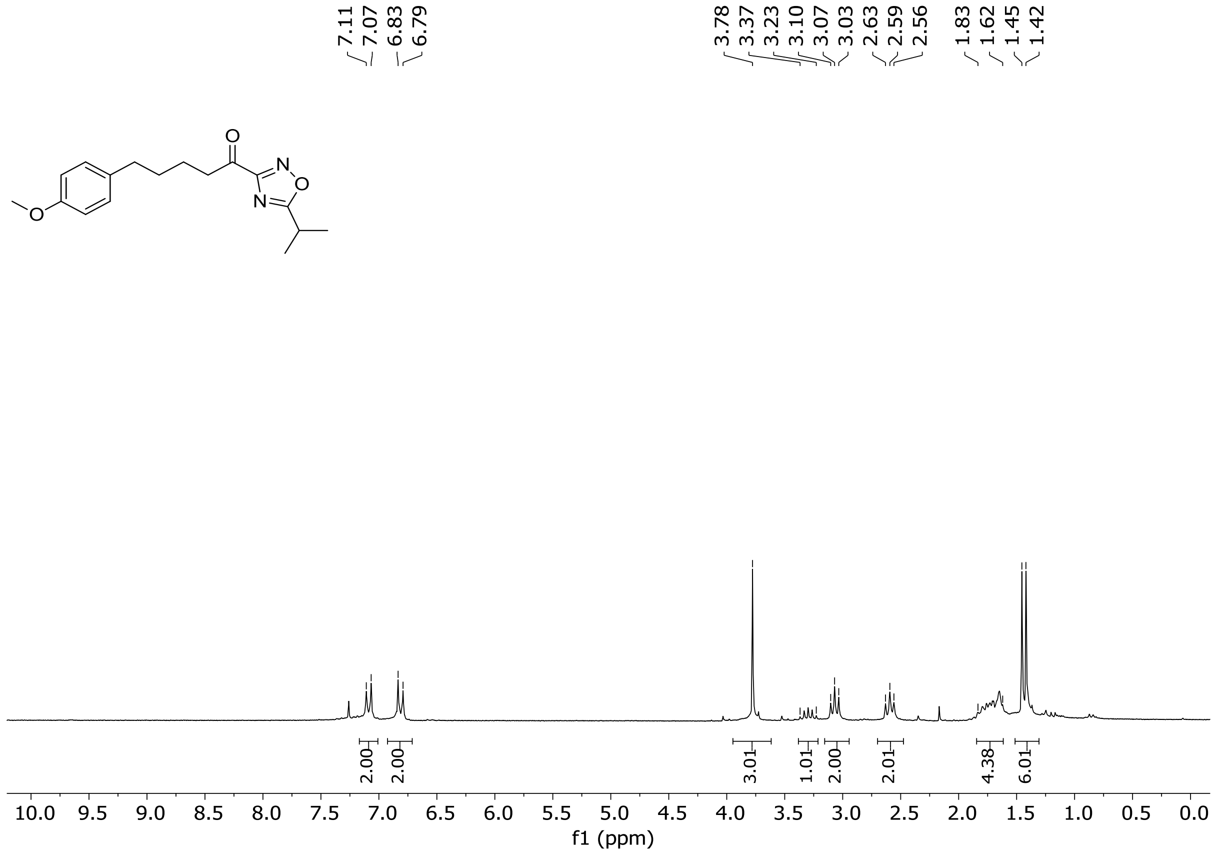
7.11
7.07
6.83
6.79
3.78
3.37
3.23
3.10
3.07
3.03
2.63
2.59
2.56
1.83
1.62
1.45
1.42
O
N
O
N
2.00
2.00
3.01
1.01
2.00
2.01
4.38
6.01
10.0 9.5 9.0 8.5 8.0 7.5 7.0 6.5 6.0 5.5 5.0 4.5 4.0 3.5 3.0 2.5 2.0 1.5 1.0 0.5 0.0
f1 (ppm)

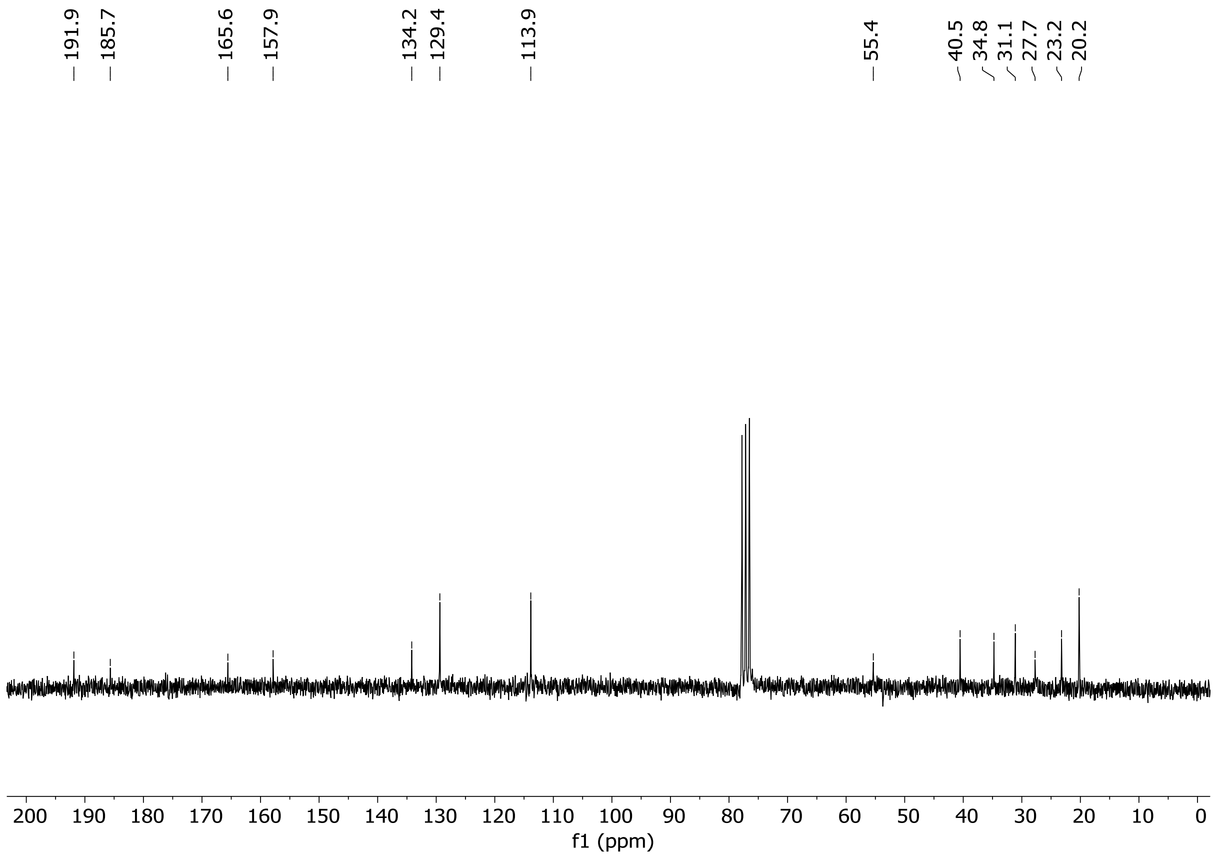
191.9
185.7
165.6
157.9
134.2
129.4
113.9
55.4
40.5
34.8
31.1
27.7
23.2
20.2
200 190 180 170 160 150 140 130 120 110 100 90 80 70 60 50 40 30 20 10 0
f1 (ppm)
